# Patient Experiences With Online Laboratory Test Presentations From Access to Activation: Systematic Review

**DOI:** 10.2196/88259

**Published:** 2026-05-29

**Authors:** Mia Liza A Lustria, Lovinta Atrinawati, Obianuju Aliche, Kyunghye Kim, Zhe He

**Affiliations:** 1 School of Information, Florida State University, 142 Collegiate Loop, Tallahassee, FL, 32306, United States, 1 8506445775; 2 Department of Quantitative Health Sciences, Mayo Clinic, Jacksonville, FL, United States; 3 Florida State University Libraries, Florida State University, Florida, United States

**Keywords:** patient portals, laboratory test results, patient engagement, health communication, health literacy, clinical workflow, health disparities, health information technology, result interpretation, patient comprehension

## Abstract

**Background:**

Federal regulations require that laboratory test results be released to patients through online portals in near–real time, often before clinicians review or contextualize them. While these policies expand transparency and affirm patients’ rights to their health information, access alone does not ensure that patients can make sense of their results or determine when and how to act. How regulatory mandates for transparency translate into appropriate engagement remains poorly understood.

**Objective:**

We aim to synthesize evidence on how patients access, interpret, and act upon laboratory test results delivered online; identify factors associated with comprehension and engagement; and evaluate how display design, clinical workflow, and patient characteristics shape engagement across the access-to-activation continuum.

**Methods:**

Following PRISMA (Preferred Reporting Items for Systematic Reviews and Meta-Analyses) 2020 guidance, we systematically searched 5 databases—Web of Science, Embase, PubMed, CINAHL, and Library, Information Science & Technology Abstracts—for English-language, peer-reviewed empirical studies published from January 2013 through April 2026, supplemented by hand searching and citation tracking. Eligible studies examined patient access to, interpretation of, or responses to laboratory results delivered through portals. Records were independently screened, appraised for methodological quality, and synthesized using thematic analysis across a 3-stage continuum: access, interpretation, and activation. The protocol was not registered.

**Results:**

Thirty-nine studies met the inclusion criteria; most were from the United States. Retrospective analyses documented increases in result viewing, secure messaging, and follow-up visits after immediate-release policies. Patients value timely access but often struggle to determine the significance of borderline values and when follow-up is warranted. Visual and textual cues can improve clarity but may also overemphasize minor deviations or inadequately signal clinically significant findings. Patients frequently monitor portals and initiate contact to confirm clinician review, indicating that increased portal activity often reflects efforts to resolve uncertainty rather than confident self-management. Disparities in access, comprehension, and confidence were observed among older adults, non–English-speaking patients, those with public insurance, and those with lower health or digital literacy. Measures of comprehension and engagement varied widely.

**Conclusions:**

Expanded online access improves transparency but does not guarantee accurate interpretation or appropriate follow-up. In contrast to prior reviews examining patient perceptions, comprehension, and presentation formats in isolation, this synthesis traces patient experiences across an access–interpretation–activation continuum, revealing how interpretive challenges and emotional responses compound across stages. Meaningful activation depends not only on display clarity but on how informational, emotional, and workflow factors shape patients’ decisions across stages. Designing for activation, therefore, requires a holistic, systems-level approach that aligns result presentation, interpretive support, and clinical workflow to signal the clinical significance of laboratory test results and guide appropriate follow-up. Such approaches must be responsive to the needs of patients across diverse literacy, language, and clinical contexts.

## Introduction

By law, patients must have immediate access to their laboratory test results through patient portals upon availability. Expanding digital access is mandated by federal regulations under the 21st Century Cures Act [1] and is a critical national objective of Healthy People 2030 [2]. The policies assert that broadening access can enhance patients’ ability to make informed decisions and strengthen their engagement in their own care. However, most portals provide little context or guidance, placing additional cognitive and emotional demands on patients.

Laboratory results are the most frequently viewed type of health data in portals. According to the Office of the National Coordinator for Health IT, over 90% of portal users view their laboratory results [3]. Yet access alone does not ensure that patients can accurately interpret or act on their test results in a timely and appropriate manner. Access without interpretive support often leads to confusion, misinterpretation, or anxiety [4-7]. These challenges are amplified among disadvantaged and higher-risk groups. Non-White, non-English-speaking, older, lower-literacy, and publicly insured patients are less likely to view their results online. Research shows that these groups tend to have lower trust in portals, greater difficulty understanding their results, and less confidence in their ability to act on them [8-12]. Limited health or digital literacy compounds these challenges further, such that those who already face barriers to care may be less likely to benefit from online access to test results [13-15].

Recognizing these gaps, the Clinical Laboratory Improvement Advisory Committee and the Agency for Healthcare Research and Quality have highlighted the need for result delivery that goes beyond timeliness to address understandability, contextualization, and equitable access [16]. The Clinical Laboratory Improvement Advisory Committee specifically encouraged the Centers for Disease Control and Prevention to create guidelines for designing more culturally and linguistically appropriate result formats [5]. Moreover, multistakeholder forums involving patients and clinicians have underscored that unclear or delayed communication contributes to diagnostic errors and missed follow-up, highlighting the risks associated with fragmented delivery systems and poorly designed portal interfaces [17].

This shift has intensified interest in understanding not only how patients access their laboratory results, but also how they interpret those results and whether this process leads to appropriate follow-up and engagement in self-management. We conceptualize this process as a continuum from access to interpretation to activation. Access concerns when and how patients receive their results and the extent to which transparency supports or overwhelms them. Interpretation involves comprehension of numerical or textual information, emotional responses to normal or abnormal findings, and judgments about urgency or need for action. Activation reflects confidence, motivation, and concrete steps taken after viewing results, including contacting clinicians, seeking additional information, or changing health behaviors.

Several recent reviews have contributed valuable insights into how patients experience and manage access to their test results through patient portals. Petrovskaya et al [18] laid an important foundation by mapping patient and clinician perspectives on the direct release of laboratory and imaging results, highlighting emotional reactions, perceived understanding, and information-seeking behavior. van der Mee et al [19] offered complementary evidence on how different presentation formats affect comprehension of numerical laboratory data. Building on this body of work, our review integrates findings from qualitative, experimental, and observational research—including surveys, retrospective analyses, and mixed-methods studies—published through 2026 to trace the full continuum from access to interpretation to activation within the evolving policy landscape shaped by the 21st Century Cures Act [20]. By aligning findings across design, workflow, and equity perspectives, we aim to provide a more integrative view of how patients encounter, make sense of, and act on their laboratory results online and underscore the importance of connecting digital tools to clinical workflows and provider communication, and establishing system-level supports for sustained engagement.

Specifically, this systematic review addresses two questions: (1) How do patients engage with and respond to online access to laboratory test results, as reflected in both portal-use behaviors and self-reported experiences? and (2) What patterns emerge across studies regarding patients’ comprehension, emotional responses, and subsequent actions after viewing laboratory test results online? It synthesizes more than a decade of empirical research and identifies recurring patterns, persistent disparities, and individual differences in experiences with online laboratory result access. It also considers their implications for the design and implementation of patient-facing systems in the era of immediate result release.

## Methods

### Study Design

This systematic review was conducted and reported in accordance with the PRISMA (Preferred Reporting Items for Systematic Reviews and Meta-Analyses) 2020 guidelines [21], with search methods reported following PRISMA-S (Preferred Reporting Items for Systematic Reviews and Meta-Analyses literature search extension [22]; see Checklist 1 for the completed PRISMA checklist).

### Search Strategy

#### Overview

We used multiple strategies to identify eligible studies. The goal was to capture a broad range of study designs and methodologies to capture patient experiences across diverse clinical and technological contexts. A research librarian (KK) developed and executed the initial comprehensive searches across Web of Science (Clarivate Analytics), Embase (Elsevier interface), PubMed (including records not yet indexed in Medline), CINAHL (EBSCOhost), and Library, Information Science & Technology Abstracts (EBSCOhost).

The search strategy was structured around two primary conceptual domains: (1) laboratory test results and related constructs (eg, laboratory values, blood tests, and patient-facing access through portals or electronic health records [EHRs]), and (2) patient interpretation and understanding, including health literacy and related constructs such as comprehension, numeracy, and readability. Search strategies were adapted across databases to account for differences in indexing systems, controlled vocabularies, and platform-specific syntax. Controlled vocabulary terms – including MeSH (Medical Subject Headings) in PubMed, Emtree in Embase, and CINAHL Headings—were supplemented with keyword-based strategies to ensure comprehensive retrieval by including potentially nonindexed records. We refined the search strategies iteratively over this study’s period by expanding keyword sets and incorporating additional controlled vocabulary terms.

Searches were limited to English-language, peer-reviewed empirical studies involving human participants and published between January 2013 and April 2026. Where database functionality permitted, we applied filters to exclude nonempirical publication types such as editorials, commentaries, and letters.

We also hand-searched key journals (eg, *JAMIA, BMC Medical Informatics and Decision Making*, *Clinical Chemistry and Laboratory Medicine*, *JMIR*, and *JMIR Medical Informatics*) and examined the bibliographies of related systematic reviews [18,19,23,24]. Two reviewers (ZH and MLAL) identified key papers from the initial search, manually reviewed their reference lists, and conducted citation tracking using Google Scholar.

The initial database searches were conducted in September 2022 and updated in July 2023, June 2024, and October 2025. We reran the refined search strategy across all databases and supplementary sources in April 2026 to ensure that the review was both comprehensive and current. Complete database-specific search strategies, including exact Boolean syntax, field tags, applied limits, and search dates, are provided in Multimedia Appendix 1 [8-14,25-56] in accordance with PRISMA-S reporting standards [22].

#### Eligibility Criteria and Initial Screening

Two authors, ZH and MLAL, developed the inclusion and exclusion criteria to identify papers eligible for full-text review. We included studies if they examined any of the following: (1) patient or caregiver perceptions or experiences accessing laboratory test results through patient portals; (2) factors associated with patients’ access to, interpretation of, or understanding of laboratory test results presented through portals or EHRs; (3) presentation styles, display formats, visualization approaches, or interface designs for viewing laboratory test results online; (4) strategies or interventions designed to improve patient access to, comprehension of, or decision-making based on online laboratory test results; (5) patient use of laboratory-test-related functions within patient portals or similar systems.

Studies were excluded if they met any of the following conditions: (1) focused solely on notification or communication of laboratory test results; (2) examined general patient portal use without a specific focus on laboratory result viewing; (3) focused primarily on the perspectives of clinicians, students, or other health professionals; (4) primarily dealt with imaging or radiology reports, or with diagnostic test results (eg, COVID-19 polymerase chain reaction tests, genomic tests, or pharmacogenomic tests); (5) did not report primary empirical data (eg, editorials, commentaries, narrative reviews, and protocols).

### Study Selection and Screening

All records identified through database searches, hand-searching of key journals, citation tracing, and screening of related systematic reviews were imported to Covidence (Veritas Health Innovation Ltd), a web-based systematic review management platform. In total, 8973 records were identified across all databases and supplementary sources following the final updated search in April 2026. Covidence automatically removed duplicate records across databases, and remaining duplicates were removed manually. Two reviewers (MLAL and ZH) independently screened titles and abstracts, followed by full-text screening of potentially relevant papers. Disagreements at either stage were resolved through discussion or, when necessary, consultation with a third reviewer. Reasons for exclusion were documented in Covidence and are summarized in the PRISMA flow diagram (see Results section).

### Quality Assessment

LA and OA independently evaluated the methodological quality and risk of bias of the 39 studies included in the final synthesis using appropriate appraisal tools. We used JBI (Joanna Briggs Institute) critical appraisal tools to assess risk of bias for randomized controlled trials [57] and quasi-experimental studies [58]. Qualitative studies were appraised using the Critical Appraisal Skills Programme qualitative studies checklist [59], survey-based studies were assessed using the Appraisal of Cross-Sectional Studies tool [60], mixed methods studies were assessed using the Mixed Methods Appraisal Tool [61], and retrospective studies were assessed using an adapted version of the Newcastle-Ottawa Scale [62].

For each study, overall risk-of-bias classifications (low, moderate, or high) were derived qualitatively based on domain-level judgments within each appraisal tool (low, some concerns, or high). We excluded studies only when critical methodological limitations precluded meaningful interpretation of the findings—ie, fundamental limitations in study design or reporting (eg, insufficient description of methods, unclear data sources, or inability to assess analytic validity). Overall judgments were based on structured reviewer evaluation rather than numeric scoring. Disagreements between reviewers were resolved through discussion and consensus. Multimedia Appendix 1 includes risk of bias tables reporting results of the quality appraisal for each study.

### Data Extraction and Synthesis

#### Overview

To guide data collection, MLAL developed and piloted a structured table to extract study characteristics and outcome domains relevant to the review objectives, including comprehension, risk interpretation, emotional responses, and activation-related behaviors. Further, 2 reviewers (LA and OA) independently extracted data using this table. Discrepancies were resolved through comparison and discussion.

#### Analysis Strategy

Following data extraction, we conducted a recursive thematic synthesis. As the included studies varied substantially in design, populations, and outcome measures, quantitative synthesis through meta-analysis was not appropriate. Moreover, key constructs such as comprehension, interpretation, emotional responses, and activation-related behaviors were operationalized in diverse and noncomparable ways, precluding calculation of a common effect size. Furthermore, no subset of studies was sufficiently comparable in terms of outcomes, measures, and study design to support a meaningful subgroup meta-analysis. The narrative synthesis was conducted and reported in accordance with guidance for synthesis without meta-analysis [63] (Checklist 2).

#### Coding and Thematic Synthesis

We developed initial codes through close reading of extracted study findings, then 2 reviewers (LA and OA) independently applied and refined these codes. Coding proceeded iteratively, with codes merged, split, or refined as patterns emerged. Discrepancies were documented and resolved through structured comparison of coded content followed by discussion. When needed, 2 additional reviewers (MLAL and ZH) reviewed disputed cases to reach consensus.

We used thematic analysis to identify recurring patterns and areas of divergence across studies. These inductively derived patterns were subsequently organized within a 3-stage continuum reflecting patients’ experiences with online laboratory test results—access, interpretation, and activation—to reflect the sequential yet interconnected nature of patients’ experiences with online laboratory test results.

### Protocol and Registration

A working protocol guided all stages of the review, and it was refined as methodological decisions were finalized. The research questions that shaped data extraction and synthesis were established prospectively and remained consistent, though the scope broadened over time in response to the evolving literature. The stage-based synthesis framework—access, interpretation, and activation—was developed iteratively during analysis and was not fully specified in the initial protocol, consistent with accepted practice in thematic synthesis where analytic frameworks commonly emerge from engagement with the data. The protocol was not publicly registered.

## Results

### Overview

**Figure 1.**
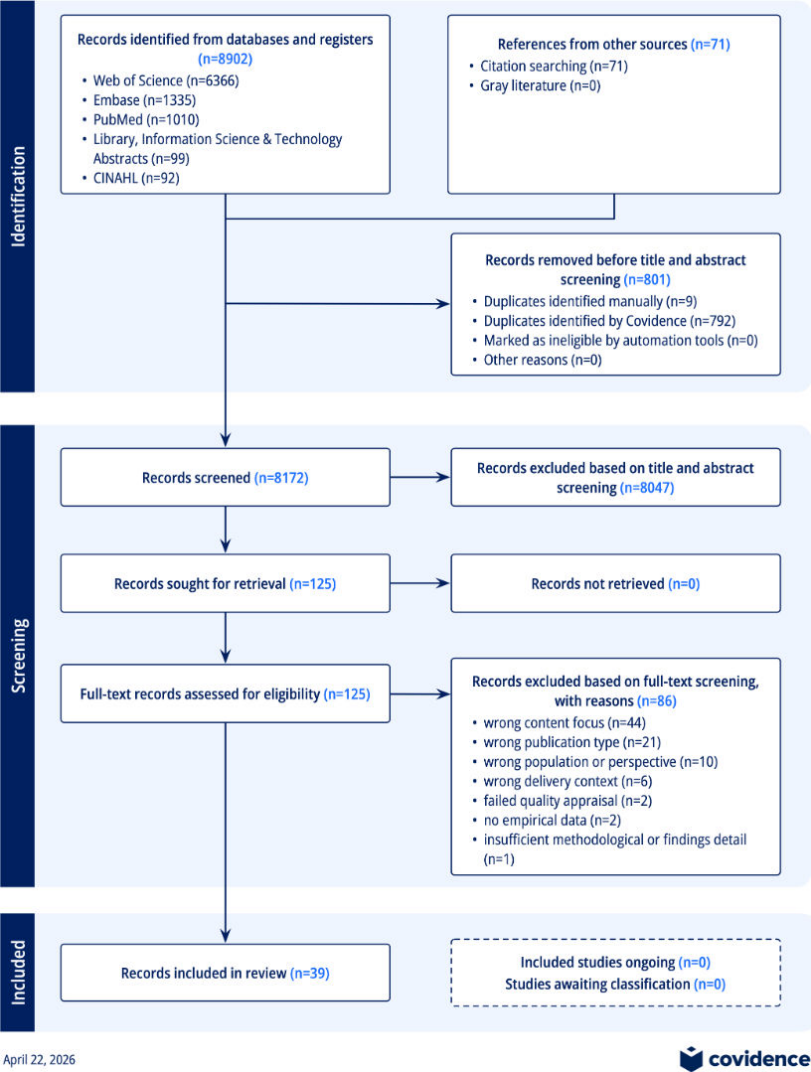
PRISMA flow diagram. PRISMA: Preferred Reporting Items for Systematic Reviews and Meta-Analyses.

Figure 1 presents the PRISMA 2020 flow diagram summarizing study identification, screening, eligibility assessment, and inclusion. Following duplicate removal, title and abstract screening, full-text review, and quality appraisal, 39 studies met the inclusion criteria and were retained for final synthesis.

### Characteristics of Included Studies

A total of 39 studies met the inclusion criteria. These represented a wide range of study designs, settings, and patient populations. Study designs included qualitative studies (n=9), retrospective analyses (n=8), survey research (n=7), experimental or quasi-experimental methods (n=8), and mixed-methods research (n=7). Most studies were conducted in the United States (n=25), followed by Canada (n=8), the Netherlands (n=5), and the United Kingdom (n=1). Table 1 includes an overview of included studies and their mapping to the access–interpretation–activation framework. (Expanded descriptions of studies, including measures and main findings, are found in Multimedia Appendix 2, Detailed Summary of Included Studies [8-14,25-56].

**Table 1.** Characteristics of included studies mapped to stages. Multimedia Appendix 2

Authors and country	Study design and purpose	Sample characteristics	Access	Interpretation	Activation
Bhalla et al [8], United States	Retrospective study: assessed changes in patient viewing of diagnostic test results via a patient portal before and after the 21st Century Cures Act.	Patients with cancer with portal-released diagnostic test results (n=44,419; 2017‐2022).	P^a^		
Christensen and Sue [55], United States	Cross-sectional survey: examined patients’ emotional responses and follow-up behaviors after viewing laboratory test results online.	Adults who viewed at least 1 laboratory test result online in the past 12 months (n=1546; median age 58 y).		P	S^b^
Foster and Krasowski [44], United States	Retrospective study: analyzed patient portal activation and diagnostic test result viewing patterns among emergency department patients.	Emergency department patients receiving laboratory or radiology tests at a university medical center (n=25,361; mean age not reported).	P		
Fraccaro et al [33], United Kingdom	Within-subjects controlled experiment: tested whether alternative patient portal display formats influence risk interpretation of laboratory test results.	Kidney transplant recipients with chronic kidney disease (n=20; mean age 52 y).		P	
Giardina et al [36], United States	Qualitative study: explored patient experiences and reactions to receiving abnormal laboratory test results through a patient portal.	Adults who accessed a patient portal and received an abnormal laboratory test result for themselves or as caregivers (n=13; age range 30‐80 y).	P		
Giardina et al [35], United States	Mixed-methods study: examined patients’ understanding, emotional responses, and follow-up actions after receiving test results via patient portals.	Adult outpatients who viewed test results through a patient portal (n=95; mean age 54.6 y).	P	P	
Hulter et al [48], Netherlands	Mixed-methods sequential explanatory study: examined patient preferences for the timing of online release of laboratory, radiology, and pathology test results through a hospital patient portal.	Hospital patient portal users with access to delayed test result release options (survey n=4592; follow-up interviews n=7; mean age not reported).	P	S	
Hulter et al [50], Netherlands	Qualitative interview study: explored patient experiences and perceived advantages and disadvantages of real-time access to test results via hospital patient portals.	Outpatients who used or did not use portals for real-time test result access (n=28; age range 16‐75 y).	P		S
Joseph et al [54], Canada	Qualitative interview study: explored how frequent users of laboratory portals interpret test results when complementary health information is presented within the same display.	Frequent users of online laboratory result portals (n=24; age range 24‐64 y).		P	
Krasowski et al [43], United States	Retrospective observational study: examined patient access to diagnostic test results through a patient portal across laboratory, pathology, and radiology settings.	Patients with diagnostic tests across outpatient, inpatient, and emergency settings over 6 months (n=59,388; mean age not reported).	P		
Lustria et al [25], United States	Cross-sectional survey study: examined patient use of patient portals to view laboratory test results and factors associated with test result comprehension.	Adults with one or more chronic conditions who used patient portals to view laboratory results (n=276; mean age 50.7 y).		P	
Mak et al [30], Canada	Cross-sectional survey study: compared turnaround time perceptions, comprehension, and anxiety between patients who accessed laboratory test results online and those who received results through traditional methods.	Adults who accessed laboratory test results via a web-based portal (n=2047) and matched non–portal users (n=1245; only age groups reported).	S	P	
McFarland et al [56], United States	Retrospective observational study: compared patient engagement with laboratory and radiology test results through an online patient portal.	Patients at an academic medical center with available electronic medical record data (n=424,422; median age 49 y).	P		
Monkman et al [49], Canada	Qualitative interview study: explored why patients use online laboratory result portals and what information they seek when reviewing their test results.	Adults with at least 2 years of experience using online laboratory result portals (n=25; age range 18‐74 y).	P		
Monkman et al [51], Canada	Qualitative interview study: examined how individuals without medical training interpret abnormal laboratory test results in mock online portals and identify potential errors and their implications.	Adults with experience accessing laboratory test results online (n=25; age range 18‐74 y).		P	
Monkman et al [42], Canada	Web-based survey: examined barriers and facilitators to using online laboratory result portals, including usability, clarity, and preferences for display features.	Adults with experience using laboratory result portals (n=30; age group ≥45 y reported).	P	P	
Monkman et al [52], Canada	Mixed-methods study: examined health consumers’ preferences for 4 online laboratory result display formats (tabular, annotated, visual, trends + contextual information)	Adults with experience accessing laboratory test results online (n=24; age range 19‐74 y).		P	
Monkman et al [53], Canada	Qualitative study: examined how adults interpret a longitudinal potassium laboratory results graph modeled on a community laboratory portal, focusing on sources of confusion, misinterpretation, and intended follow-up actions.	Adults with experience accessing laboratory test results online (n=24; age range 19‐74 y).		P	S
Morrow et al [32], United States	Randomized experimental study: tested how different patient portal message formats (standard, verbal, graphical, and video-enhanced) influence older adults’ understanding of numeric test results, including memory for values, interpretation of risk, and intentions to engage in self-care behaviors.	Older adults viewing cholesterol and diabetes test results (n=144; mean age 71.9 y).		P	S
Nystrom et al [29], United States	User-centered design study: developed and evaluated a patient-facing prototype for reviewing laboratory test results through a patient portal.	Adult patients (n=14; mean age 43 y; age range 25‐73 y).		P	S
Pillemer et al [39], United States	Mixed-methods study: examined how direct release of laboratory test results through patient portals influences patient engagement and health care usage.	Patient portal users at an integrated health system during 1 year (n=6368 survey respondents; mean age 51.8 y).	P		
Robinson et al [31], Canada	Qualitative interview study: explored why patients use patient portals to access laboratory test results and how this access influences comprehension, engagement, and care behaviors.	Adult primary care patients who actively used a portal’s laboratory results feature (n=21; age range 60‐69 y).	P	S	
Scherer et al [26], United States	Web-based experimental study: tested whether substituting clinically appropriate goal ranges for standard reference ranges improves understanding of glycated hemoglobin test results.	US adults with and without type 2 diabetes recruited via a national web-based panel (n=6766; mean age 49.1 y).		P	
Schultz and Alderfer [37], United States	Qualitative study: explored caregivers’ preferences and experiences related to receiving pediatric cancer test results through online patient portals.	Caregivers of children with cancer at a pediatric hospital (n=19; average age 40.4 y).	P		
Solomon et al [27], United States	User-centered design study: developed and iteratively tested prototype visualizations to improve patient comprehension and interpretation of laboratory test results in patient portals.	Adults with diabetes or family members of individuals with diabetes (n=18; median age range 50‐59 y).		P	
Steitz et al [47], United States	Large multisite survey study: examined patient and caregiver perceptions of immediate release of test results through online patient portals, including perceived anxiety, engagement, and preferences.	Patients and caregivers who accessed test results through a patient portal (n=8139; median age 64 y).	P		
Steitz et al [12], United States	Retrospective observational study: examined patient portal use while awaiting laboratory test results, focusing on repeated access behavior and patient-initiated messaging.	Adult patients with outpatient laboratory tests and active patient portal accounts at an academic medical center (portal logs for 3,29,317 users; age not reported).	P		
Steitz et al [46], United States	Interrupted time series design: evaluated whether releasing laboratory test results in a patient-friendly educational format through a patient portal was associated with a reduction in patient-initiated messaging.	Adult outpatients who received laboratory test results via an Epic MyChart (Epic Systems Corporation) portal at a single academic medical center (n=205,139; mean age 51.0 y; 2024).	P		
Struikman et al [38], Netherlands	Web-based experimental study: tested whether enhanced patient portal designs for viewing blood test results influence patient health engagement.	Adults recruited from a national consumer panel who viewed fictional blood test results (n=487; mean age 52.8 y).			P
Talboom-Kamp et al [10], Netherlands	Cross-sectional survey study: examined patient experiences and self-efficacy after viewing laboratory test results through an online patient portal with explanatory text and visuals.	Patient portal users who viewed laboratory test results and completed a web-based survey immediately afterward (n=354; mean age 58.5 y).			P
Tossaint-Schoenmakers et al [9], Netherlands	Cross-sectional survey study: examined how patient characteristics influence attitudes toward an online patient portal for communicating laboratory test results.	Adults who accessed blood test results through a web-based patient portal (n=748; mean age 58.5 y).			P
Turer et al [45], United States	Retrospective observational study: examined real-time patient portal use for viewing diagnostic test results during emergency department visits.	Adult emergency department encounters with at least 1 diagnostic test result (60,314 encounters representing 31,164 unique patients; age not reported).	P		
Wood et al [40], United States	Retrospective prepost observational study: examined changes in the timing of patient viewing of diagnostic test results following implementation of immediate release policies.	Patients with laboratory or imaging test results at an academic medical center before and after policy implementation (204,605 patients; mean age 44.3 y).	P		
Zhang et al [13], United States	Sequential explanatory mixed-methods study: explored patient challenges, needs, and emotional responses when interpreting laboratory test results through patient portals.	Adults who had previously accessed laboratory test results through a patient portal (survey n=203; interview n=13; only age groups reported).		P	
Zhang et al [41], United States	User-centered, mixed-methods design study: developed and evaluated a patient-facing system prototype to improve communication of laboratory test results through patient portals.	Adults who recently accessed laboratory test results through a patient portal (survey n=203; interview n=13; mean age 43.5 y).		P	
Zhong et al [11], United States	Retrospective observational study: examined associations between patient portal use patterns and subsequent health care usage and appointment adherence.	Adult primary care patients with and without portal use over a multiyear period (matched samples n=4312 users and n=4024 nonusers; only age groups reported).	P		
Zikmund-Fisher et al [28], United States	Web-based experimental study: examined patients’ ability to identify out-of-range laboratory test values and perceived urgency when viewing standard tabular test results.	US adults (n=1817; mean age 54.2 y).		P	
Zikmund-Fisher et al [14], United States	Web-based experimental study: tested whether graphical displays help patients distinguish between urgent and nonurgent deviations in laboratory test results.	US adults (n=1620; mean age 48.9 y).		P	
Zikmund-Fisher et al [34], United States	Web-based experimental study: examined whether adding harm anchors to visual displays of laboratory test results reduces perceived urgency and worry for near-normal values.	US adults (n=1618; mean age 48.8 y).		P	

a P**: **primary analytical focus of the study within the patient experience continuum.

b S**: **secondary analytical focus.

### Main Outcome Variables and Measurement

#### Comprehension

Measurement strategies varied substantially across studies. Comprehension was assessed both objectively and subjectively. Overall, about one-third of studies (n=12) used objective comprehension or behavior metrics, while the remainder relied primarily on self-report. Objective measures included value-interpretation tasks assessing accuracy in identifying whether results were within or outside reference or goal ranges [25-29]. Some studies asked participants to self-rate their understanding and confidence in interpreting laboratory results [9,13,25,30] or to evaluate comprehension of medical terms and reference ranges [31]. Further, 1 study measured verbatim memory for exact numerical values and gist memory for the qualitative meaning or risk implications of results to examine how older adults understand and respond to portal-based test results [32].

#### Risk Interpretation and Perceived Urgency

Risk interpretation captures the cognitive evaluation of the seriousness or health implications of a laboratory result, whereas perceived urgency reflects the perceived need for timely action in response to that information. Both were not measured consistently in the same way, and several studies assessed them jointly rather than as separate outcomes. Risk interpretation was typically measured through risk-appraisal tasks asking participants to judge the clinical seriousness of an abnormal laboratory value—whether it required follow-up or no action based on perceived severity [33]. Additionally, 1 study used broader risk-perception items adapted from Garcia-Retamero and Cokely [64], focusing on perceived likelihood of complications [32]. Perceived urgency focused on how quickly participants believed they should respond after viewing results. It was typically assessed using Likert-type items evaluating urgency of taking action or contacting a clinician or by asking participants about intended timing of response, such as whether they would contact a clinician immediately, wait, or take no action) [14,28,34].

#### Emotional Responses

Emotional responses to viewing laboratory results online were typically elicited through surveys or semistructured interviews. Only a few studies used validated affective scales [30,35]. Mák et al [30] assessed anxiety using the validated Global Anxiety–Visual Analog Scale [65], which measures emotions from “no anxiety” to “worst imaginable anxiety” on a 100 mm line.

#### Patient Activation and Health Engagement

Patient activation was assessed in several ways, mostly through self-report [35-37]. Struikman et al [38] used the Patient Health Engagement, a 5-item scale, measuring patients’ psychological readiness and involvement in managing their health across 4 progressive phases—capturing emotional, cognitive, and behavioral dimensions of health engagement. Further, 2 studies [9,10] used the 10-item motivation and confidence to act subscale of the eHealth Impact Questionnaire [66] to determine the extent to which eHealth technology influences patients’ confidence in taking health-related actions. Giardina et al [35] used the Patient Activation Measure [67]—a 10-item validated scale used to assess participants’ engagement and capacity for health self-management.

#### Engagement With Laboratory Test Features in Patient Portals

Retrospective studies captured objective measures of portal engagement (eg, frequency of portal logins and laboratory test viewing), capturing both initial access and downstream follow-up behaviors [8,39,40]. User log analyses also captured behaviors such as patient-initiated provider contacts, follow-up appointments, and no-shows [11,12,37,39].

#### Portal Acceptability, Usability, and Satisfaction

Design-focused studies captured outcomes related to usability, user satisfaction, and visual engagement with laboratory result interfaces. Usability was commonly assessed using the system usability scale [29,41,68]. The net promoter score [69], a single-item measure of patients’ likelihood to recommend the portal experience, was used in 1 study to measure satisfaction [42]. A few studies used the 13-item information and presentation subscale of the eHealth Impact Questionnaire to evaluate users’ perceptions of the clarity, usefulness, and trustworthiness of health information [9,10]. Furthermore, 1 study used eye tracking to measure fixation count, average fixation duration, and dwell time to capture how participants processed laboratory result displays [33].

### Results of Thematic Analysis

The following section organizes the results of the thematic synthesis along a 3-stage continuum—access, interpretation, and activation—synthesizing evidence on facilitators and barriers to portal use, timing and immediate release practices, comprehension, risk appraisal, emotional responses, and subsequent engagement behaviors.

#### Access

Access refers to the structural and contextual conditions under which laboratory test results are made available through patient portals.

##### Access Before and After Policy Changes

Several large-scale retrospective studies compared portal access before and after changes in laboratory result release policies [1]. Krasowski et al [43] analyzed portal log data from 59,388 patients in 2016 and found that only 30% of outpatient laboratory results were viewed through the patient portal. A follow-up emergency department (ED)–focused analysis conducted 3 years later showed only modest change, with 8.9% of ED-ordered laboratory results viewed through the patient portal [44]. Among those that were accessed, 53.3% were viewed within 72 hours of release, while nearly 20% were not viewed until 2 weeks or more after becoming available.

Following policy changes, multiple studies documented significant increases in portal-based laboratory result viewing patterns, particularly in outpatient settings. Turer et al [45] observed an increase in ED result viewing from 5% to 15% between April 2021 and April 2022, even though the portal was not actively promoted. Wood et al [40] analyzed portal log data from over 204,000 users, comparing two 10-month periods (February to December 2020 vs February to December 2021). They found that the proportion of test results viewed within 1 day rose from 22% to 31% among adult users and from 15% to 33% among pediatric proxy users following the policy change. Similarly, Bhalla et al [8] analyzed log data of 44,000 oncology patients and observed a drop in median viewing time from 77.0 hours to 6.4 hours after the policy was implemented. By 2022, a total of 75% of laboratory results were viewed by patients before their providers. Steitz et al [46] corroborated this pattern in a 2024 sample of over 829,000 outpatient results, finding that 69.4% were accessed by patients before their ordering clinician.

Default settings and notification features were associated with higher rates of laboratory result viewing. A retrospective analysis of portal log data showed increased real-time test result viewing among ED patients following changes in default release settings that enabled immediate push notifications [45].

##### System-Logged Patient Behaviors Following Immediate Result Release

Portal log analyses provide additional insight into how patients interact with results once they become available. Pillemer et al [39] found that patients who viewed automatically released laboratory results had higher rates of office visits and telephone calls than those who received manually released results. Steitz et al [12] analyzed portal log and secure messaging data from over 1.2 million outpatient test encounters. They found that patients who frequently refreshed pages and received routine test results were more likely to send a secure message to their provider soon after viewing the results than those who received high-sensitivity test results. Steitz et al [46] found that 17.4% of reviewed outpatient results prompted a patient-initiated message within 24 hours of release. Patient-initiated messaging was not meaningfully reduced when supplementary patient-friendly educational materials were provided.

Zhong et al [11] tracked engagement among 4312 portal users at a university hospital and found that patients who actively used the laboratory feature had higher rates of office visits and calls to their providers and had lower no-show rates (30%‐60%) compared to matched nonusers.

##### Patient-Reported Preferences, Benefits, and Barriers Related to Portal-Based Laboratory Result Viewing

Preferences for immediate versus delayed access varied across populations and clinical contexts. In a national survey of US portal users, most preferred to receive results as soon as they became available—even before their provider had reviewed them [47]. In a retrospective study, most Dutch portal users set preferences for a 1-day delay for laboratory test result release [48]. Follow-up interviews revealed that some users later changed their preferences to shorter delays, citing the advantages of greater transparency and time to prepare for upcoming appointments. Those who had opted for longer delays said they changed their preference settings because they found the information hard to understand or lacking in sufficient detail. Timing preferences also differed based on result sensitivity. While many patients supported immediate access to abnormal results, some preferred that serious or highly concerning results be communicated verbally before appearing in the portal [36]. Schultz and Alderfer [37] found that caregivers of pediatric cancer patients preferred to be informed about abnormal test results directly by their child’s doctor and viewed electronic delivery as mainly supplementary.

In Monkman et al [49], participants found online access reassuring and helpful for monitoring their chronic diseases, medications, and treatments. Some noted that online access improves patient safety by facilitating early detection of abnormalities and prompt communication with their doctors. In 2 studies, caregivers and patients acknowledged that portal access reduces the burden on clinic staff and frees up clinical resources by reducing unnecessary appointments and allowing physicians to prioritize more urgent cases [31,37].

Despite these reported advantages, studies also identified barriers to using portals for laboratory result viewing. Common challenges included difficulty locating test results within portals, confusion when multiple systems were in place, and uncertainty about which platforms to use. In a survey of adults with chronic conditions, many participants who had viewed laboratory results online still preferred to discuss results directly with their clinicians or only wanted to be notified if their results were abnormal [25]. Giardina et al [36] interviewed 13 US adults—patients reported that results often lacked adequate explanation and contextual cues, reducing the perceived usefulness of immediate access.

##### Emotional Responses to Immediate and Unmediated Release

Immediate portal access to laboratory results can evoke a range of emotional responses. In Giardina et al [36], patients who received abnormal results were significantly more likely to report concern, confusion, or anxiety than those receiving normal results. Some participants described worry even when results were within range, particularly when no clinician interpretation was provided. Pillemer et al [39] used a mixed-methods design combining survey data, interviews, and EHR usage logs from 6368 patient portal users. Some participants were worried when abnormal results were released automatically without physician explanations. One interviewee thought she had cancer after seeing an abnormal test result on a weekend and was only reassured when she was able to talk to her doctor the next business day.

Emotional reactions were not uniformly negative. In a large cross-sectional survey, Steitz et al [47] found that many participants indicated that viewing results directly reduced worry compared to waiting for clinician notification. For some patients, immediate access alleviated the stress associated with delayed communication. Qualitative findings from Hulter et al [48] and Hulter et al [50] further illustrate this tension. While some participants felt overwhelmed when encountering unexpected or sensitive information online, others valued early access because it shortened the waiting period and provided a sense of control before clinician discussion.

##### Disparities in Portal Access and Laboratory Result Viewing

Studies consistently documented disparities in those who enrolled in patient portals and those who used them to view laboratory test results, with differences falling along the lines of race, ethnicity, insurance type, language preference, gender, and care setting. Zhong et al [11] reported enrollment disparities in a hospital-based sample. Nonusers were more often Black, Hispanic, male, unmarried, or publicly insured. Portal adopters were more often older than an age of 30 years and had more telephone encounters with providers. Missed appointments were more common among nonusers.

Portal log analyses documented parallel disparities in viewing behavior. Bhalla et al [8] reported that only 33% of Black patients viewed their laboratory results after they were released, compared to 51% of White and 58% of Asian patients. Wood et al [40] found that White, English-speaking, privately insured patients—particularly those seen in outpatient settings—were more likely to view results within 1 day of release. Viewing rates were also lower among older adults, non-White and non-English-speaking patients, and those with public or no insurance.

Krasowski et al [43] and Foster and Krasowski [44] reported disparities in viewing behavior across care settings. Outpatient laboratory results were accessed more frequently (around 30%) than those receiving inpatient or emergency care (under 10%). Access was more limited for parents of adolescent patients, reflecting proxy access restrictions in some settings.

Lustria et al [25] identified predictors of laboratory result access. White race and higher eHealth literacy were positively associated with viewing results through portals, while having a college degree was associated with lower odds of access after controlling for other factors.

##### Recommended Design Features to Support Access

Participants across studies recommended design improvements to make laboratory result portals easier to navigate and more inclusive. Clean visual structure and simpler layouts were recurring themes. In 1 study, participants reported difficulty scanning results within long tables and recommended persistent column headers and visually highlighting abnormal values to improve readability [51].

Formatting and usability issues related to mobile access were noted across several studies [27,41,42]. Participants in Solomon et al [27] indicated the need for larger fonts and minimal visual clutter, particularly for mobile viewing. In another study, participants noted that existing portal designs do not translate well to small screens and recommended mobile apps with more intuitive navigation [42].

Accessibility concerns were especially salient for older adults and individuals with visual or cognitive impairments. Suggested features included screen-reading options, streamlined navigation, and assistive buttons operable by keyboard or touch. One participant in the study by Zhang et al [13] emphasized the importance of designing the portal so that “grandma can understand it.” Another user in Robinson et al [31] suggested including a short onboarding tutorial to help less technology-savvy patients understand how to navigate the portal and locate test results.

### Interpretation

This stage reflects the cognitive and affective work required to translate numerical data into meaningful information.

#### Identifying and Interpreting Abnormal Results

A central task in interpretation involves determining whether a test value falls outside the reference range and understanding what that deviation signifies. The absence of explanatory context was the most consistently reported barrier to understanding. In a mixed-methods study, Giardina et al [35] reported that although many interviewees said they understood the information, several struggled to interpret specific values. One participant, for example, was unsure whether the term “positive” indicated a favorable or unfavorable result. Lustria et al [25] assessed comprehension of hypothetical laboratory panels in a survey of 276 US adults. While most participants scored well on objective tests, they rated their own understanding of the values as low and preferred physician explanation—particularly when values fell outside the normal range.

Across multiple studies, participants reported difficulty determining whether values were normal or abnormal when results appeared in dense tables without plain-language explanations or clear visual indicators [13,29,42,49,51,52]. In an experiment with 1817 US adults, Zikmund-Fisher et al [28] found that only 51% correctly identified a moderately elevated hemoglobin A_1c_ (HbA_1c_) value (8.4%) as outside the reference range when presented in a standard table format. Many participants also struggled to rate glycemic control accurately or determine whether they would contact their physician. Monkman et al [51] asked experienced portal users to review a mock laboratory result on an existing portal display; even among these active users, many failed to recognize abnormal results when values were not clearly flagged or contextualized. In iterative design workshops, Nystrom et al [29] observed confusion around HDL (high-density lipoprotein) cholesterol values, with many participants assuming that higher HDL levels were unfavorable—reflecting a broader expectation that higher numbers are undesirable, even though higher HDL is typically associated with lower cardiovascular risk.

Experimental studies further demonstrated that the format in which laboratory results are displayed can substantially influence the accuracy of interpretation. In a randomized study with older adults, Morrow et al [32] found that verbally and video-enhanced displays improved participants’ ability to interpret the meaning of results compared to graphical formats, although recall of exact numerical values did not improve. Extending this to trend displays, Monkman et al [53] found that 18 of 24 participants expressed confusion about at least 1 graph feature, and only 2 said they would seek urgent follow-up for an out-of-range value.

Not all visualization styles improved comprehension. Displays incorporating multiple reference ranges, dense formatting, or layered graphical elements often increase cognitive burden and reduce interpretive accuracy. Scherer et al [26] found that comprehension was highest when visualized test results emphasized a single, clearly defined goal range. Simpler visuals that directly stated what a result meant and whether action was needed were rated more favorably, whereas layered displays with multiple reference zones and graphical indicators reduced comprehension. In user-centered evaluations of mock portal reports, participants described lengthy layouts and technical language as overwhelming, particularly when abnormal values were not clearly emphasized [51]. Similarly, Joseph et al [54] found that supplementing trend graphs with educational content was sometimes perceived as confusing or misaligned with the test result itself.

#### Appraising Risk and Urgency

Risk interpretation and perceived urgency represent related but distinct dimensions of interpretation. As many studies assessed these constructs using overlapping measures—asking participants to judge either the seriousness of a result or how quickly action was needed—the findings are reported together here.

Display formats influenced how patients judged the seriousness of results and the perceived need for immediate follow-up. Visual formats generally reduced overestimation of urgency for near-normal results compared with standard tables. Zikmund-Fisher et al [14] found that participants viewing standard tabular displays were more likely to judge mildly abnormal alanine transaminase and creatinine values as urgent than those viewing visual formats. Visual formats improved differentiation between mildly and clearly abnormal values but did not substantially alter perceptions of extreme results. Scherer et al [26] found that showing only goal ranges—rather than both goal and standard reference ranges—was associated with more accurate judgments of urgency. Zikmund-Fisher et al [34] found that “harm-anchor” displays reduced perceived urgency for near-normal results and decreased intentions to contact a clinician or seek hospital care for those values, without affecting responses to more extreme abnormalities.

Visualizations were not uniformly effective in reducing misclassification of risk severity. Fraccaro et al [33] tested 3 result formats (baseline, contextualized horizontal bars, or grouped) with kidney transplant recipients across low-, medium-, and high-risk scenarios. Participants had the most difficulty identifying medium-risk scenarios and frequently selected follow-up actions that did not align with recommended care. Although eye-tracking data showed longer viewing time for contextualized formats, none of the 3 displays significantly improved interpretation accuracy [33]. Morrow et al [32] similarly found that participants in the graphically enhanced condition rated lower-risk results as more serious than those in the verbally enhanced, video-enhanced, and standard conditions, with a similar pattern observed for borderline-risk results. In a think-aloud study of a portal-style potassium graph, Monkman et al [53] reported that only 2 of 24 participants said they would seek urgent follow-up for an out-of-range value, while most preferred to look up information, follow up later, or were not in a hurry to contact a provider.

#### Individual Differences in Interpretation

Risk appraisal and comprehension varied based on different characteristics. Zikmund-Fisher et al [28] found that participants with higher numeracy were more likely to correctly identify significantly abnormal HbA_1c_ values and reported stronger intentions to contact their physician. Higher health literacy was associated with a lower likelihood of follow-up for mildly elevated values. Zikmund-Fisher et al [14] reported that participants with higher health and graphical literacy were better able to distinguish between mildly and highly abnormal results, particularly when results were presented using visual displays. Zhang et al [13] reported that more participants with higher health literacy and technology proficiency said the portal helped them understand their results and feel supported. Lustria et al [25] found that older adults and frequent portal users were significantly more likely to score higher on objective comprehension tests, although many self-reported difficulty understanding their results and preferred to have doctors explain them.

#### Affective Responses During Result Interpretation

The action of interpreting test results presented online can evoke a range of negative and positive emotional reactions. The evidence reported here includes both studies of patient reactions to actual test results and studies using hypothetical or simulated scenarios.

Negative emotions such as anxiety, worry, and confusion were commonly reported when results were abnormal, unexpected, or presented without a clear explanation. Concern and distress were strongest when terminology was unfamiliar or when patients were uncertain whether a clinician had reviewed the result [13,31,35,36,39]. In Giardina et al [35], several participants reported anxiety or confusion even with normal results—particularly when no interpretation or guidance was included. Similarly, in a survey comparing patient portal users with nonusers, Mák et al [30] found that users who were unclear about the need for follow-up were more likely to report higher anxiety.

Participants in the study by Robinson et al [31] described physician comments accompanying laboratory results as clarifying and reassuring, particularly when comments confirmed that the result had been reviewed and did not require further action. Although uncertainty frequently elicited anxiety, reassurance was also reported when results were clearly contextualized. In 1 study, among participants with chronic conditions, those who accessed results online were significantly less likely to report high anxiety [30].

#### Presentation Features That Shape Emotional Responses

In experimental and user-centered studies using hypothetical or simulated test results, presentation styles—including language and terminology, visual cues, and other enhancements—also evoked different emotions. Zhang et al [41] found that emotionally charged phrasing such as “not optimal” was perceived as alarming. Participants preferred neutral, explanatory language placed directly alongside test results. Scherer et al [26] examined emotional responses to hypothetical HbA_1c_ results presented in 3 formats: as a table, a 2-color number line, or a block-style number line. They found that block-style displays using color-coded diagnostic categories (eg, red for “high” or “abnormal”) were significantly rated as more discouraging than simpler number lines or tables. Similarly, Solomon et al [27] found that red flags and warning icons elicited mixed reactions—some found them helpful; others described them as anxiety-inducing.

#### Design Recommendations to Support Interpretation

To make portal-released laboratory results easier to understand, more trustworthy, and more reassuring, patients frequently emphasized the need for plain-language explanations to help interpret unfamiliar or abnormal results. They suggested providing summaries that clearly state whether a value was within a healthy range and what it meant. One participant in the study by Pillemer et al [39] said it would have helped to have “a note that said normal/abnormal or something like that.” In Monkman et al [42], users similarly asked for short, condition-specific explanations that clarified the purpose of each test and its relevance to their treatment.

Patients also expressed interest in receiving tailored and contextualized explanations, personalized resource links, and tailored prompts [41]. In 1 study, patients wanted explanations tailored based on users’ literacy levels and options to receive either basic summaries or advanced content. Similarly, Monkman et al [42] found that embedded explanations tailored to the patient’s medical context could help clarify the relevance and meaning of specific laboratory values. Monkman et al [52] found that many participants wanted to combine color-coded summaries with trend graphs and tabular details and preferred being able to switch between visual and tabular formats, indicating a desire to tailor not only explanations but also the format of laboratory result displays to their needs. In Zhang et al [13], some participants proposed integrating artificial intelligence to provide contextualized explanations based on individual health data.

To increase confidence in laboratory results, participants in 1 study recommended cues indicating provider oversight—such as checkmarks signaling that their doctor had reviewed their results [50] or some kind of indication from their doctor about the need for follow-up [31]. Some participants said that labels such as “your doctor is pleased,” and simple text overlays such as “you are here” or “your doctor wants you here” could provide reassurance and guide interpretation, especially when paired with graphical displays [27,31]. Lastly, patients often expressed the need for vetted, high-quality health information alongside laboratory results to help them interpret their results [41,54].

### Activation

The activation stage refers broadly to patients’ readiness, confidence, and actions taken in response to viewing laboratory results online. Across included studies, related constructs such as engagement, motivation, behavioral intention, and self-efficacy were used inconsistently to capture this domain.

#### Online Access and Behavioral Intentions

Patients commonly described reflecting on their health, monitoring conditions, and making small behavior changes in response to viewing laboratory results online [29,31,49,50]. Hulter et al [50] reported that online access helped Dutch patients prepare for appointments, identify errors, and engage more actively in health decisions. Monkman et al [49] found that participants routinely scanned for abnormal values and tracked trends over time, describing laboratory result access as a tool for self-care and personal accountability. In Nystrom et al [29], 64% of patients said viewing their results motivated them to change their diet or exercise, and over a third intended to follow up with their provider. Portal users interviewed by Robinson et al [31] also reported that online access improved their ability and motivation to monitor their health more closely, adhere to their medications, and make lifestyle changes.

#### Information-Seeking and Follow-Up Actions

Across studies, patients frequently reported searching for additional information, preparing for medical visits, or contacting providers after receiving results online [13,35,39,55]. Christensen and Sue [55] found that patients engaged in a range of follow-up information-seeking behaviors after viewing their results, including talking with others (21%), searching for information in the portal (20%), creating graphs (19%), and looking up information online (18%). These actions were more common when providers had not clearly communicated how results would be shared. Pillemer et al [39] found that patients often used early access to look up related terms, track chronic conditions, or prepare questions—sometimes reaching out even when results were not clinically significant.

#### Factors Influencing Motivation to Act

Several experiments using hypothetical or simulated scenarios found that laboratory result presentation formats influenced patients’ intentions to act or engage in their care [14,32,34,38]. In the studies by Zikmund-Fisher [14,34], patients shown mildly or near-normal abnormal results in visual formats were more likely to report they would delay follow-up or take no immediate action compared to those who viewed tabular displays. Morrow et al [32] observed that graphically enhanced presentations were associated with stronger intentions to adhere to prescribed medications, particularly for lower-risk results, although visual format had less consistent effects on intentions to change diet or exercise.

Struikman et al [38] assessed how different laboratory result display formats influenced patients’ emotional, cognitive, and motivational readiness to engage in their own care using a shortened version of the validated Patient Health Engagement scale. Engagement scores declined significantly after viewing abnormal results without context. When visuals and explanations were included, engagement remained stable for normal and partially abnormal results and declined only slightly for abnormal ones.

Talboom-Kamp et al [10] found a strong positive correlation between perceived portal usability—particularly clarity, ease of understanding, and trust—and self-efficacy. Similarly, participants who rated the portal’s information and presentation more favorably reported higher motivation and confidence to act on laboratory results (*r*=0.77, *P*<.001).

#### Desired Support for Follow-Up and Engagement

Patients often advocated for features that would help them take informed next steps. Many expressed the need for automated alerts when new results became available. In Monkman et al [42], participants recommended adding follow-up support features, such as automated notifications when results were available and links to additional resources or options to message providers. Others stressed that these alerts should do more than announce availability; participants in Giardina et al [35] suggested pairing notifications with short explanations and recommendations about what to do next.

Some patients recommended tools to support follow-up visits with their providers. Participants in Zhang et al [41] recommended adding an “ask questions” button that would help clarify test results and help them prepare personalized questions for follow-up visits. This feature would allow them to save questions directly within the portal and generate question lists for clinical visits. This, they felt, would help structure conversations and remind them about key concerns they need to discuss. In another study, patients also suggested portals should include brief follow-up instructions, lifestyle suggestions, and access to trusted health sources [13].

## Discussion

### Principal Findings

This review synthesized evidence from 39 studies on how patients engage with and respond to online access to laboratory test results. Across a diverse body of evidence, patients’ engagement is characterized by increased access, but also by substantial variability in how results are understood and acted upon. Studies consistently show that access does not reliably translate into accurate interpretation or appropriate action, particularly when results are released without accompanying clinical interpretation. Patients frequently struggle to determine the clinical significance of results, especially when values are borderline, unexpected, or lack a clear contextual explanation. These interpretive challenges evoke emotional responses such as anxiety, reassurance, or uncertainty, which in turn shape subsequent actions, including seeking additional information, contacting providers, or taking no action. Overall, access alone does not ensure meaningful engagement; patients’ ability to comprehend and respond to their results varies and is shaped by both the characteristics of the results and systematic differences in patient capacity and access to support.

Prior reviews have typically examined patient portal use, comprehension, or presentation strategies in isolation, often focusing on a single outcome domain, mixing patient and clinician perspectives, or aggregating different types of test results such as laboratory, imaging, and pathology findings [18,19,24]. In contrast, this synthesis brings together complementary forms of data from qualitative studies, large-scale analyses of real-world portal use, survey research, controlled experiments, and mixed-methods designs to examine how patients engage with laboratory results across the full trajectory from access to interpretation to action. Log data document patterns of real-world engagement, survey research identifies the patient characteristics and contextual factors associated with comprehension and follow-up, qualitative inquiry explains the interpretive and emotional processes underlying these patterns, and experimental studies demonstrate how design features shape understanding and decision-making. This integrated perspective provides a more complete account of patient engagement, highlighting how challenges emerge and interact across stages rather than occurring as isolated barriers.

The following sections interpret these findings across the 3 stages, examining how informational, emotional, and workflow factors interact to shape patient engagement at each point along the continuum.

### Access: Transparency, Immediacy, and Workflow Alignment

Patients often described valuing having their laboratory results available online and finding it more convenient than waiting for phone calls or scheduled visits, particularly for routine or anticipated findings [36,47,49,55]. For many, immediate access signals transparency and supports a sense of control over their care [47,48,50]. At the same time, patients expected that clinicians would still review results and provide reassurance, especially when findings are abnormal, unexpected, or difficult to interpret [25,36,37]. Access, in other words, is valued not as a replacement for clinician communication but as an earlier entry point into that relationship.

Our synthesis suggests that this dual expectation exposes a structural misalignment between how access is implemented and how responsibility for follow-up is allocated and signaled. The results may be released automatically into portals while clinician review and communication occur asynchronously, leaving patients unsure whether anyone has assessed their results or will proactively reach out [12,36]. In this context, immediate access shifts interpretive and logistical work from clinicians and staff to patients, who must decide when to wait and when to initiate contact [11,12,39]. Requests for clearer interpretive cues and guidance about next steps [13,41], as well as for visible indicators of clinician review [12,36], can be understood as attempts to rebalance these responsibilities rather than as resistance to immediate access.

At a policy level, immediate-release regulations have broadened the possibility of access but have not produced equitable realization of that access across populations or care settings [8,40,45]. Across the included studies, viewing rates were lower among non-White and non–English-speaking patients, older adults, people with lower health or digital literacy, and those with public insurance [8,11,25,40]—groups who may have the most to gain from timely information yet face the greatest barriers to portal use. These patterns likely reflect structural constraints (eg, language support, connectivity, enrollment practices, or trust in institutions) more than a lack of interest in results, indicating that equity in the access stage depends as much on addressing these upstream conditions as on refining portal interfaces themselves.

### Interpretation: Sensemaking Under Uncertainty

#### Overview

While portal-based delivery of laboratory results has expanded transparency, it also amplifies the interpretive work patients must do to determine what their numbers mean for their health.

#### Signaling Relevance and Proportionality

To make sense of a laboratory result, patients must judge whether deviations from standard ranges are meaningful, and that judgment depends heavily on how the deviation is signaled. In portal displays, values appear alongside reference ranges, color indicators, flags, and graphical elements that denote whether a test value falls outside expected limits. Experimental studies show that these formats shape perceived significance, but not always proportionately: some clarify trends and contextualize variation, whereas others amplify minor fluctuations or highlight borderline values in ways that overstate their clinical importance [14,26,34].

For example, a value only slightly outside a reference range may be interpreted as urgent when displayed in red, while more clinically meaningful patterns may be obscured by dense or layered interfaces [26,27]. Displays that are overly complex can increase cognitive load and reduce confidence in interpretation. By contrast, overly simplified cues can shift perceptions of seriousness and urgency in directions that do not always align with clinical meaning—especially when patients must interpret results on their own [32,33].

Moreover, these preferences and effects are not uniform. Some users prefer detailed, layered information, while others find it overwhelming. What enhances clarity for one group can increase confusion or anxiety for another [13,42,49,52].

#### Contextualization and Tailoring

Across qualitative and survey studies, patients report that they do not simply want to know whether a value falls within a reference range; they want to understand what the results mean relative to their health condition, treatment, and broader health trajectory [13,25,49]. In response, designers of patient-facing systems are often urged to “contextualize” and “tailor” laboratory results, but these terms are frequently used interchangeably. Although both are intended to help patients make sense of their results, contextualization and tailoring are implemented in different ways and serve related, but distinct functions within laboratory result communication.

Contextualization refers to providing information that clarifies the clinical meaning of a result, for example, by explaining reference ranges, using categorical risk labels, or adding brief explanatory text [26,28]. In contrast, tailoring involves designing messages for a specific individual based on assessed characteristics related to the outcome of interest (eg, beliefs, health literacy, numeracy, or motivation), with the goal of increasing perceived personal relevance and influencing how information is interpreted and acted upon [70]. When used appropriately, tailoring has been shown to promote deeper processing and increase how seriously information is taken [71,72]. In patient-facing laboratory result systems, this is most often operationalized through computer tailoring (ie, algorithm-based or data-driven messaging), where individual data are used to generate tailored information or guidance via mechanisms such as personalization, feedback, and content adaptation [73,74].

These distinctions, however, are rarely made explicit. Many recommendations invoke “tailoring” without specifying what and how much individual details are beneficial, which elements should be adapted, or how tailoring should operate in clinically ambiguous situations. As systems, including artificial intelligence–driven tools, become capable of generating highly individualized explanations, there is a tendency to assume that greater specificity—whether through contextualizing information or tailored messaging—will improve understanding. Yet additional detail can introduce noise, elevate perceived importance, or shift interpretive weight without resolving uncertainty. When these strategies are implemented primarily through layering additional data or explanations, they risk introducing complexity and cognitive load without improving interpretive clarity or reliably supporting more meaningful engagement. The challenge, then, is not simply to provide more contextualization or more intensive tailoring, but to calibrate how contextual information and tailored messages are introduced so that they sharpen clinical meaning without overwhelming patients or unduly inflating the perceived importance of test results.

#### Emotion, Meaning Making, and Design

Laboratory results are often viewed in moments of uncertainty, and patients commonly feel worried, confused, or overwhelmed by raw numbers and jargon [13,31,35,39]. These emotions are heightened in the absence of contextual framing [8,30]. Interface elements intended to clarify meaning, such as color coding, flags, summary labels, and risk terms (“abnormal,” “suboptimal,” or “high risk”), influence whether a deviation feels urgent or routine; bold alerts and red blocks may heighten alarm for marginal findings, while muted or cluttered layouts can obscure patterns that warrant concern [14,26,27,34,41].

Despite extensive documentation of these emotional responses, the literature offers limited guidance on how systems should deliberately calibrate them. Human-computer interaction research provides a useful lens for this challenge. Emotional design involves deliberately structuring visual layout, language, and interaction cues to shape how users feel in moments of uncertainty [75,76]. Potential strategies include neutral summary statements that precede numerical details, restrained and well-explained use of color and flags, visible indicators that clinicians will review results within a specified timeframe, and concise descriptions of typical next steps. Such features may help assure patients that they are not alone in making sense of their results. Without this kind of emotional calibration, increased transparency may lead to confusion or distress rather than informed understanding.

### Activation: Engagement Beyond Access

Policy narratives tend to equate transparency and immediate access with automatic gains in patient engagement. In practice, activation is inseparable from the interpretive and emotional work patients perform after viewing their results. Patients’ decisions about whether to watch and wait, seek reassurance, or request urgent care are shaped by how clearly results are explained, how deviations from normal are framed numerically and visually, and how worried or reassured patients feel in the moment [14,32,34,38]. Experimental and user-centered studies show that visualization formats clarifying the magnitude and direction of deviation can reduce unnecessary worry or intentions to seek urgent care for near-normal values, yet they may also blunt vigilance for higher-risk results, including cases where clearly abnormal trends are interpreted as nonurgent [14,34,53]. In this way, online result systems are not neutral delivery channels—they may shape the kinds of downstream actions that patients see as appropriate.

At the same time, what appears in log data as “activation” may reflect gaps and misalignments in clinical workflows rather than purely patient-driven engagement. Across the included studies, actions such as viewing results repeatedly, sending secure messages, or scheduling appointments may reflect attempts to resolve residual uncertainty rather than confident self-management [13,35,39,55]. In this sense, “more action” is not inherently better; meaningful activation is better characterized as calibrated and proportionate follow-up that matches the clinical seriousness of the result and the patient’s goals, and supports patients’ preparedness for shared decision-making.

Additionally, when results are released before clinicians have reviewed or communicated them, patients often send multiple messages, call offices, or seek in-person visits to confirm that results have been seen and to clarify their meaning [35,39]. These compensatory behaviors suggest that activation is a property of the broader sociotechnical system: result displays, notification rules, and response pathways either converge to scaffold timely, collaborative follow-up or leave patients to improvise their own paths to clarification. Designing activation-supportive systems, therefore, requires coupling patient-facing interpretive aids with predictable, clearly signposted routes to clinician review and shared decision-making.

Finally, current operationalizations of activation tend to obscure important inequities. When activation is inferred primarily from portal behaviors or self-initiated contacts, it may be overestimated for already-advantaged populations and underestimated for patients whose capacity to act is constrained by language, literacy, or structural factors. The same groups that face barriers to portal access and comprehension (eg, non-White and non–English-speaking patients, older adults, people with lower health or digital literacy, and those with public insurance) are less likely to view results promptly and to feel confident about what to do next [8,11,25,40]. Similar patterns observed across the continuum suggest that this may have cascading effects—patients who face barriers to access have fewer opportunities to encounter and make sense of their results, and those who struggle to interpret results are less equipped to take meaningful action.

### Limitations

This review has several limitations. First, it focused on peer-reviewed, English-language studies and did not include gray literature such as preprints, dissertations, or non–peer-reviewed conference proceedings. This approach may have excluded early-stage innovations and practice-based insights from nonacademic or non–English-speaking settings, increasing the risk of publication bias.

The evidence base is also geographically concentrated. Few studies examined experiences in non-US health systems or among patients with limited connectivity or portal access. The findings underrepresent the needs of less digitally engaged groups, individuals with lower literacy or limited English proficiency, and patients who do not access portals. This limits insight into how expectations for result delivery, interpretation, and follow-up may differ across health systems and levels of digital access.

Limitations within the primary studies also influence the strength of the conclusions. Researchers defined and operationalized key constructs inconsistently and rarely used validated instruments, making it difficult to compare findings across studies or determine whether observed differences reflect true variation in patient responses or are artifacts of measurement. This also constrained conclusions about the impact of specific design features, as similar outcomes were often labeled or measured in incompatible ways. More broadly, this reflects a conceptual gap in the field: without shared definitions and validated measures, the literature cannot accumulate in a way that supports robust, generalizable conclusions. Progress will require the development and adoption of common, validated measures that can support meaningful comparison across studies, populations, and health system contexts.

Many studies relied on self-report measures, introducing recall and social desirability biases and limiting insight into how patients interpreted or acted on laboratory results in practice. Researchers also frequently used hypothetical test scenarios rather than real clinical encounters. Although these designs allowed controlled comparisons of presentation features, they did not fully capture how patients responded when results were personally relevant, emotionally salient, and embedded within ongoing care.

Most experimental studies used small, convenience samples, often drawn from populations already engaged with digital health tools. These samples provide limited insight into how findings extend to broader and more diverse patient populations, particularly those with lower digital literacy, limited access to technology, or different expectations for interacting with health information. Studies also rarely examined whether design features function differently across patients with varying health histories, goals, or prior knowledge. Without attention to this heterogeneity, it remains unclear under what conditions specific presentation strategies are effective or for whom they are most beneficial.

Finally, most studies assessed immediate or short-term responses to laboratory result presentations, while relatively few examined longer-term behavioral or clinical outcomes. This emphasis on proximal outcomes limits the ability to determine whether improvements in comprehension or reassurance translate into sustained changes in follow-up behavior, decision-making, or engagement in care over time.

### Conclusions

Patients who access laboratory test results through online portals are not simply retrieving information; they are navigating a sequence of decisions—whether to attend to a result, how to interpret it, and what to do next—often in the absence of timely clinical guidance. The evidence synthesized here shows that expanding access has shifted engagement earlier in the care process but has not ensured that patients can effectively use the information. Instead, challenges compound across stages: results are encountered without sufficient context, interpretation remains uncertain, and expectations for follow-up are often unclear. As a result, patient responses vary widely and are not consistently aligned with clinical need. This review advances prior work by demonstrating that these gaps are not isolated issues of comprehension or interface design but reflect a broader misalignment between how results are delivered, when they are encountered, and the support available to patients as they move from access to action.

Improving patient engagement, therefore, requires changes across portal design, clinical workflows, and institutional policies, rather than isolated enhancements to any single component. In practice, this includes embedding interpretive context at the point of result delivery, clearly signaling clinician review and follow-up expectations, and providing accessible pathways for patients to seek clarification or act. These supports must be designed for diverse populations and real-world use at scale. Differences in health literacy, language proficiency, digital access, and experience with the health system shape not only whether patients view results but also how they interpret and respond to them. Without deliberate design and implementation strategies—such as language access, graduated interpretive support (eg, layered explanations, visual cues, and thresholds for action), and outreach beyond already engaged users—expanded transparency may reinforce existing disparities by placing a greater interpretive burden on those least equipped to manage it. Translating access into meaningful, equitable engagement, therefore, depends on designing systems that support patients across stages while reducing, rather than redistributing, this burden.

## Supplementary material

10.2196/88259 Multimedia Appendix 1 Search strategy, and risk of bias tables.

10.2196/88259 Multimedia Appendix 2 Detailed summary of included studies.

10.2196/88259 Checklist 1 PRISMA 2020 checklist.

10.2196/88259 Checklist 2 SWiM checklist.

## Abbreviations

ED: emergency department
EHR: electronic health record
HbA_1c_: hemoglobin A_1c_
HDL: high-density lipoprotein
JBI: Joanna Briggs Institute
MeSH: Medical Subject Headings
PRISMA: Preferred Reporting Items for Systematic Reviews and Meta-Analyses
PRISMA-S: Preferred Reporting Items for Systematic Reviews and Meta-Analyses Literature Search Extension
