# Supplementary material for: Patient Experiences With Online Laboratory Test Presentations From Access to Activation: Systematic Review
Source: J Med Internet Res. 2026 May 29;28:e88259. doi: 10.2196/88259 (PMC13222930; doi:10.2196/88259)
Supplement: Multimedia Appendix 1 [file jmir-v28-e88259-s001.pdf]

This appendix consolidates the reporting and quality assessment materials for this systematic review in accordance with PRISMA 2020 and PRISMA-S guidelines. It includes the complete database-specific search strategies (with platform syntax, applied limits, and search dates), and detailed risk-of-bias tables for all included studies.

Table of Contents

Search Strategies 2
- Database: Web of Science 2
- Database: Embase 2
- Database: PubMed 3
- Database: Library, Information Science & Technology Abstracts (LISTA) 3
- Database: CINAHL 3

Quality Assessment - Risk of Bias Tables 5
- **Table S1.** Risk of Bias Table for Mixed Methods Studies 5
- **Table S2.** Risk of Bias Table for Experimental Research Studies 5
- **Table S3.** Risk of Bias Table for Survey Research Studies 6
- **Table S4.** Risk of Bias Table for Qualitative Research Studies 6
- **Table S5.** Risk of Bias Table for Retrospective or Portal-Based Research Studies 7

List of Quality Appraisal Tools Used 7

References 8

Search Strategies

Below are the complete database-specific search strategies used for this review in accordance with PRISMA-S reporting standards. Searches were developed in collaboration with a professional research librarian and adapted for each database platform to accommodate differences in indexing systems, controlled vocabularies (e.g., MeSH, Emtree, and CINAHL Headings), proximity operators, and syntax requirements.

The initial database searches were conducted in September 2022 and updated in July 2023, with additional searches conducted in June 2024 and October 2025. Search strategies were iteratively refined across these stages through expansion of controlled vocabulary and keyword synonym sets, as well as through review of retrieval results and identified studies. The refined strategy was rerun across all databases and sources in April 2026 to ensure that the review was comprehensive and current. Searches included English-language, peer-reviewed studies involving human subjects published between January 2013 and April 2026.

Search strategies combined controlled vocabulary terms with expanded keyword and synonym sets across the primary conceptual domains (laboratory test results, patient portals/electronic health records, patient understanding, and result presentation/visualization). All retrieved records were exported and imported into Covidence, where duplicate citations were identified and removed prior to screening. identification methods included backward and forward citation tracking, hand searching of key journals, and review of reference lists from relevant articles.

Limits and Filters

Where supported by the database platform, searches were limited to English-language studies involving human subjects and to publications from January 2013 to April 2026. Additional publication-type filters (e.g., to prioritize empirical or original research articles) were applied selectively where available to reduce retrieval of non-empirical materials (e.g., editorials, commentaries); however, final inclusion was determined through manual screening based on predefined eligibility criteria.

Database: Web of Science

- **Platform:** Clarivate
- **Date searched:** April 15, 2026
- **Results:** 6,366 records
- **Search string:** (TS=("laboratory test result*" OR "lab test result*" OR "laboratory result*" OR "lab result*" OR "blood test result*" OR "lab value*" OR "laboratory value*" OR "blood test*" OR "clinical laboratory*") AND TS=(interpret* OR "health literacy" OR literac* OR numeracy OR readabilit* OR "plain language" OR notif* OR "reference range*" OR "normal range*" OR graph* OR infographic* OR "display format*" OR "presentation format*" OR "data visualization" OR chart* OR visual*))

Database: Embase

- **Platform:** Elsevier
- **Date searched:** April 15, 2026
- **Results:** 1,335 records
- **Search string:** (‘clinical laboratory test' OR 'laboratory test result*':ti,ab OR 'lab test result*':ti,ab OR 'laboratory result*':ti,ab OR 'lab result*':ti,ab OR 'blood test result*':ti,ab OR 'blood test*':ti,ab OR 'clinical laboratory*':ti,ab OR 'lab value*':ti,ab OR 'laboratory value*':ti,ab OR 'patient portal*':ti,ab OR 'electronic health record*':ti,ab OR 'personal health record*':ti,ab OR mychart:ti,ab) AND (comprehen*:ti,ab OR underst*:ti,ab OR interpret*:ti,ab OR 'health

literacy':ti,ab OR numeracy:ti,ab OR readabilit*:ti,ab OR 'plain language':ti,ab OR notif*:ti,ab) AND (visual*:ti,ab OR graph*:ti,ab OR infographic*:ti,ab OR 'reference range*':ti,ab OR 'normal range*':ti,ab OR 'display format*':ti,ab OR 'presentation format*':ti,ab OR 'data visualization':ti,ab OR chart*:ti,ab)

Database: PubMed

- Platform: PubMed
- **Date searched:** April 15, 2026
- **Results:** 1,010 records
- **Search string:** ("Clinical Laboratory Techniques"[MeSH Terms] OR "Blood Chemical Analysis"[MeSH Terms] OR "Patient Portals"[MeSH Terms] OR "Electronic Health Records"[MeSH Terms] OR "medical records systems, computerized"[MeSH Terms] OR "Health Literacy"[MeSH Terms] OR "Data Visualization"[MeSH Terms] OR "laboratory test result*"[Title/Abstract] OR "lab test result*"[Title/Abstract] OR "laboratory result*"[Title/Abstract] OR "lab result*"[Title/Abstract] OR "blood test result*"[Title/Abstract] OR "blood test*"[Title/Abstract] OR "clinical laboratory*"[Title/Abstract] OR "lab value*"[Title/Abstract] OR "laboratory value*"[Title/Abstract] OR "patient portal*"[Title/Abstract] OR "electronic health record*"[Title/Abstract] OR "personal health record*"[Title/Abstract] OR "mychart"[Title/Abstract]) AND ("comprehen*"[Title/Abstract] OR "underst*"[Title/Abstract] OR "interpret*"[Title/Abstract] OR "Health Literacy"[Title/Abstract] OR "literac*"[Title/Abstract] OR "numeracy"[Title/Abstract] OR "readabilit*"[Title/Abstract] OR "plain language"[Title/Abstract] OR "notif*"[Title/Abstract]) AND ("visual*"[Title/Abstract] OR "graph*"[Title/Abstract] OR "infographic*"[Title/Abstract] OR "reference range*"[Title/Abstract] OR "normal range*"[Title/Abstract] OR "display format*"[Title/Abstract] OR "presentation format*"[Title/Abstract] OR "Data Visualization"[Title/Abstract] OR "chart*"[Title/Abstract])

Database: Library, Information Science & Technology Abstracts (LISTA)

- Platform: EBSCO
- **Date searched:** April 15, 2026
- **Results:** 99 records
- **Search string:** TX("laboratory test result*" OR "lab test result*" OR "laboratory result*" OR "lab result*" OR "blood test result*" OR "lab value*" OR "laboratory value*" OR "blood test*" OR "clinical laboratory*") AND TX(comprehen* OR underst* OR interpret* OR "health literacy" OR literac* OR numeracy OR readabilit* OR "plain language" OR notif* OR "reference range*" OR "normal range*" OR visual* OR graph* OR infographic* OR "display format*" OR "presentation format*" OR "data visualization" OR chart*)

Database: CINAHL

- Platform: EBSCO
- **Date searched:** April 15, 2026
- **Results:** 92 records
- Search string:

(

XB ("laboratory test result*" OR "lab test result*" OR "laboratory result*" OR "lab result*" OR "blood test result*" OR "lab value*" OR "laboratory value*")

)

AND

(

MH "Health Literacy"

OR XB ("health literacy" OR literac* OR numeracy OR readabilit* OR "plain language" OR notif* OR comprehen* OR underst* OR interpret*)

)

AND

(

MH "Patient Portals"

OR XB ("patient portal*" OR "patient portals" OR "personal health record*" OR "online access" OR "online record*" OR "electronic health record*" OR "EHR" OR "EMR" OR "test result portal*" OR "lab result portal*" OR visual* OR graph* OR infographic* OR "reference range*" OR "normal range*" OR "display format*" OR "presentation format*" OR "data visualization" OR chart*)

)

Quality Assessment - Risk of Bias Tables

Table S1. Risk of Bias Table for Mixed Methods Studies

Mixed Methods Studies	MM Rationale (justification for mixed methods)	Integration (components effectively integrated)	Interpretation (outputs adequately interpreted)	Divergence (inconsistencies addressed)	Quality Adherence (meets standards for each method)	Overall Risk of Bias
Giardina, Baldwin et al [1]	Low	Low	Low	Some concerns	Low	Moderate
Hulter, Langendoen et al [2]	Low	Low	Low	Some concerns	Low	Moderate
Monkman, MacDonald et al [3]	Low	Low	Low	Some concerns	Low	Moderate
Nystrom, Singh et al [4]	Low	Some concerns	Low	Low	Low	Moderate
Pillemer, Price et al [5]	Low	Low	Low	Some concerns	Low	Moderate
Zhang, Citardi et al [6]	Low	Low	Low	Low	Low	Low
Zhang, Kmoth et al [7]	Low	Low	Low	Low	Low	Low

Table S2. Risk of Bias Table for Experimental Research Studies

Experimental Studies	Type of Experiment	Selection and Sampling	Allocation & Comparability	Confounding Control	Outcome Measurement	Incomplete Data and Attrition	Selective Reporting	Contextual Bias or Other	Overall Risk of Bias
Fraccaro, Vigo et al [8]	Quasi	Some concerns	Low	Some concerns	Low	Low	Low	Some concerns	Moderate
Morrow, Azevedo et al [9]	RCT	Some concerns	Low	Some concerns	Low	Low	Low	Some concerns	Moderate
Scherer, Witteman et al [10]	RCT	Some concerns	Low	Low	Low	Low	Low	Some concerns	Moderate
Steitz, Guide et al [11]	Quasi	Low	Low	Some concerns	Low	Low	Low	Some concerns	Moderate
Struikman, Bol et al [12]	Quasi	Some concerns	Low	Some concerns	Low	Low	Low	Some concerns	Moderate
Zikmund-Fisher, Exe et al [13]	RCT	Some concerns	Low	Low	Low	Low	Low	Some concerns	Moderate
Zikmund-Fisher, Scherer et al [14]	RCT	Some concerns	Low	Low	Low	Low	Low	Some concerns	Moderate
Zikmund-Fisher, Scherer et al [15]	RCT	Some concerns	Low	Low	Low	Low	Low	Some concerns	Moderate

Table S3. Risk of Bias Table for Survey Research Studies

Cross-Sectional Survey Studies	Study Design (appropriate design, sample justified)	Sampling Strategy (population defined, frame, selection)	Measurement Validity (instruments validated)	Response Rate/Bias (non-response addressed)	Data Analysis (statistical methods, consistency)	Reporting (results presented, conclusions justified)	Overall Risk of Bias
Christensen and Sue [16]	Low	Low	Some concerns	Low	Low	Low	Moderate
Lustria, Aliche et al [17]	Low	Some concerns	Low	Some concerns	Low	Low	Moderate
Mak, Smith Fowler et al [18]	Low	Some concerns	Low	Some concerns	Low	Low	Moderate
Monkman, Griffith et al [19]	Low	Some concerns	Low	Some concerns	Low	Low	Moderate
Steitz, Turer et al [20]	Low	Some concerns	Low	Some concerns	Low	Low	Moderate
Talboom-Kamp, Tossaint-Schoenmakers et al [21]	Low	Some concerns	Low	High	Low	Low	High
Tossaint-Schoenmakers, Kasteleyn et al [22]	Low	Some concerns	Low	High	Low	Low	High

Table S4. Risk of Bias Table for Qualitative Research Studies

Qualitative Studies	Methodology (appropriate design)	Sampling (recruitment strategy)	Data Collection (methods appropriate)	Analysis Rigor (researcher reflexivity + rigorous analysis)	Findings Clarity (clear statement of findings)	Overall Risk of Bias
Giardina, Modi et al [23]	Low	Some concerns	Low	Some concerns	Low	Moderate
Hulter, Weggelaar-Jansen et al [24]	Low	Some concerns	Low	Low	Low	Moderate
Joseph, Monkman et al [25]	Low	Some concerns	Low	Low	Low	Moderate
Monkman, Griffith et al [26]	Low	Low	Low	Some concerns	Low	Moderate
Monkman, MacDonald et al [27]	Low	Some concerns	Low	Some concerns	Low	Moderate
Monkman, Schmit et al [28]	Low	Some concerns	Low	Some concerns	Low	Moderate
Robinson, Reed et al [29]	Low	Low	Low	Some concerns	Low	Moderate
Schultz and Alderfer [30]	Low	Low	Low	Low	Low	Low
Solomon, Scherer et al [31]	Low	Some concerns	Low	Some concerns	Low	Moderate

Table S5. Risk of Bias Table for Retrospective or Portal-Based Research Studies

Retrospective Studies	Representativeness of the study sample	Sample size	Assessment of the outcome(s)	Overall Risk of Bias
Bhalla, Prasad et al [32]	Low	Low	Low	Low
Foster and Krasowski [33]	Some concerns	Low	Low	Moderate
Krasowski, Grieme et al [34]	Low	Low	Low	Low
McFarland, Huang et al [35]	Low	Low	Some concerns	Moderate
Steitz, Turer et al [36]	Low	Low	Low	Low
Turer, Martin et al [37]	Some concerns	Low	Low	Moderate
Wood, Pham et al [38]	Low	Low	Low	Low
Zhong, Park et al [39]	Low	Low	Low	Low

List of Quality Appraisal Tools Used

- *Mixed Methods Studies:* Hong, Q. N., Fàbregues, S., Bartlett, G., Boardman, F., Cargo, M., Dagenais, P., Gagnon, M.-P., Griffiths, F., Nicolau, B., O'Cathain, A., Rousseau, M.-C., Vedel, I., & Pluye, P. (2018). The mixed methods appraisal tool (MMAT) version 2018 for information professionals and researchers. Education for Information, 34, 285–291. https://doi.org/10.3233/EFI-180221
- *Cross-Sectional Survey Studies:* Downes, M. J., Brennan, M. L., Williams, H. C., & Dean, R. S. (2016). Development of a critical appraisal tool to assess the quality of cross-sectional studies (AXIS). *BMJ Open*, 6(12), e011458. https://doi.org/10.1136/bmjopen-2016-011458
- *Qualitative Research Studies:* Long, H. A., French, D. P., & Brooks, J. M. (2020). Optimising the value of the Critical Appraisal Skills Programme (CASP) tool for quality appraisal in qualitative evidence synthesis. *Research Methods in Medicine & Health Sciences*, 1(1), 31–42. https://doi.org/10.1177/2632084320947559
- *Quasi-Experiments*: Barker, T. H., Habibi, N., Aromataris, E., Stone, J. C., Leonardi-Bee, J., Sears, K., Hasanoff, S., Klugar, M., Tufanaru, C., Moola, S., & Munn, Z. (2024). The revised JBI critical appraisal tool for the assessment of risk of bias for quasi-experimental studies. *JBI Evid Synth*, *22*(3), 378–388. https://doi.org/10.11124/JBIES-23-00268
- *Randomized Controlled Trials*: Barker, T. H., Stone, J. C., Sears, K., Klugar, M., Tufanaru, C., Leonardi-Bee, J., Aromataris, E., & Munn, Z. (2023). The revised JBI critical appraisal tool for the assessment of risk of bias for randomized controlled trials. *JBI Evid Synth*, *21*(3), 494–506. https://doi.org/10.11124/JBIES-22-00430
- *Retrospective Research Studies:* Carra, M. C., Romandini, P., & Romandini, M. (2025). Risk of bias evaluation of cross-sectional studies:

References

1. Giardina TD, Baldwin J, Nystrom DT, Sittig DF, Singh H. Patient perceptions of receiving test results via online portals: A mixed-methods study. J Am Med Inform Assoc. 2018 Apr 1;25(4):440–6. PMID: 29240899. doi: 10.1093/jamia/ocx140.
2. Hulter P, Langendoen W, Pluut B, Schoonman GG, Luijten R, van Wetten F, et al. Patients' choices regarding online access to laboratory, radiology and pathology test results on a hospital patient portal. PloS One. 2023;18(2):e0280768. PMID: 36735739. doi: 10.1371/journal.pone.0280768.
3. Monkman H, MacDonald L, Joseph AL, Lesselroth BJ. Tabular, annotated, visual, or trends + contextual information? Preferences for online laboratory results displays. Stud Health Technol Inform. 2024 Jan 25;310:1041–5. PMID: 38269973. doi: 10.3233/SHTI231123.
4. Nystrom DT, Singh H, Baldwin J, Sittig DF, Giardina TD. Methods for patient-centered interface design of test result display in online portals. EGEMS (Wash DC). 2018 Jun 26;6(1):15. PMID: 30094287. doi: 10.5334/egems.255.
5. Pillemer F, Price RA, Paone S, Martich GD, Albert S, Haidari L, et al. Direct release of test results to patients increases patient engagement and utilization of care. PloS One. 2016;11(6):e0154743. PMID: 27337092. doi: 10.1371/journal.pone.0154743.
6. Zhang Z, Citardi D, Xing A, Luo X, Lu Y, He Z. Patient challenges and needs in comprehending laboratory test results: Mixed methods study. J Med Internet Res. 2020 Dec 7;22(12):e18725. PMID: 33284117. doi: 10.2196/18725.
7. Zhang Z, Kmoth L, Luo X, He Z. User-centered system design for communicating clinical laboratory test results: Design and evaluation study. JMIR Hum Factors. 2021 Nov 25;8(4):e26017. PMID: 34842529. doi: 10.2196/26017.
8. Fraccaro P, Vigo M, Balatsoukas P, van der Veer SN, Hassan L, Williams R, et al. Presentation of laboratory test results in patient portals: Influence of interface design on risk interpretation and visual search behaviour. BMC Med Inform Decis Mak. 2018 Feb 12;18(1):11. PMID: 29433495. doi: 10.1186/s12911-018-0589-7.
9. Morrow D, Azevedo RFL, Garcia-Retamero R, Hasegawa-Johnson M, Huang T, Schuh W, et al. Contextualizing numeric clinical test results for gist comprehension: Implications for EHR patient portals. J Exp Psychol Appl. 2019 Mar;25(1):41–61. PMID: 30688498. doi: 10.1037/xap0000203.
10. Scherer AM, Witteman HO, Solomon J, Exe NL, Fagerlin A, Zikmund-Fisher BJ. Improving the understanding of test results by substituting (not adding) goal ranges: Web-based between-subjects experiment. J Med Internet Res. 2018 Oct 19;20(10):e11027. PMID: 30341053. doi: 10.2196/11027.
11. Steitz BD, Guide A, Rodriguez K, Kripalani S, Aher CV, Craig KS, et al. Patient-friendly test results and patient-initiated messaging among adult outpatients. JAMA Network Open. 2025;8(11):e2543879–e. doi: 10.1001/jamanetworkopen.2025.43879.
12. Struikman B, Bol N, Goedhart A, van Weert JCM, Talboom-Kamp E, van Delft S, et al. Features of a patient portal for blood test results and patient health engagement: Web-based pre-post experiment. J Med Internet Res. 2020 Jul 20;22(7):e15798. PMID: 32706704. doi: 10.2196/15798.
13. Zikmund-Fisher BJ, Exe NL, Witteman HO. Numeracy and literacy independently predict patients' ability to identify out-of-range test results. J Med Internet Res. 2014 Aug 8;16(8):e187. PMID: 25135688. doi: 10.2196/jmir.3241.

14. Zikmund-Fisher BJ, Scherer AM, Witteman HO, Solomon JB, Exe NL, Tarini BA, et al. Graphics help patients distinguish between urgent and non-urgent deviations in laboratory test results. J Am Med Inform Assoc. 2017 May 1;24(3):520–8. PMID: 28040686. doi: 10.1093/jamia/ocw169.
15. Zikmund-Fisher BJ, Scherer AM, Witteman HO, Solomon JB, Exe NL, Fagerlin A. Effect of harm anchors in visual displays of test results on patient perceptions of urgency about near-normal values: Experimental study. J Med Internet Res. 2018 Mar 26;20(3):e98. PMID: 29581088. doi: 10.2196/jmir.8889.
16. Christensen K, Sue V. Viewing laboratory test results online: Patients' actions and reactions. J Participat Med. 2013 Oct. 3;5:e38.
17. Lustria MLA, Aliche O, Killian MO, He Z. Enhancing patient engagement and understanding: Is providing direct access to laboratory results through patient portals adequate? JAMIA Open. 2025 Apr 2025;8(2):ooaf009. PMID: 40130170. doi: 10.1093/jamiaopen/ooaf009.
18. Mak G, Smith Fowler H, Leaver C, Hagens S, Zelmer J. The effects of web-based patient access to laboratory results in British Columbia: A patient survey on comprehension and anxiety. J Med Internet Res. 2015 Aug 4;17(8):e191. PMID: 26242801. doi: 10.2196/jmir.4350.
19. Monkman H, Griffith J, MacDonald L, Lesselroth B. Consumers' needs for laboratory results portals: Questionnaire study. JMIR Hum Factors. 2023 Jun 12;10:e42843. PMID: 37307049. doi: 10.2196/42843.
20. Steitz BD, Turer RW, Lin CT, MacDonald S, Salmi L, Wright A, et al. Perspectives of patients about immediate access to test results through an online patient portal. JAMA Netw Open. 2023 Mar 1;6(3):e233572. PMID: 36939703. doi: 10.1001/jamanetworkopen.2023.3572.
21. Talboom-Kamp E, Tossaint-Schoenmakers R, Goedhart A, Versluis A, Kasteleyn M. Patients' attitudes toward an online patient portal for communicating laboratory test results: Real-world study using the eHealth impact questionnaire. JMIR Form Res. 2020 Mar 4;4(3):e17060. PMID: 32024632. doi: 10.2196/17060.
22. Tossaint-Schoenmakers R, Kasteleyn M, Goedhart A, Versluis A, Talboom-Kamp E. The impact of patient characteristics on their attitudes toward an online patient portal for communicating laboratory test results: Real-world study. JMIR Form Res. 2021 Dec 17;5(12):e25498. PMID: 34927593. doi: 10.2196/25498.
23. Giardina TD, Modi V, Parrish DE, Singh H. The patient portal and abnormal test results: An exploratory study of patient experiences. Patient Exp J. 2015 Spring;2(1):148–54. PMID: 28345018.
24. Hulter P, Weggelaar-Jansen A, Ahaus K, Pluut B. Patient discourses on real-time access to test results via hospital portals: A discourse analysis of semistructured interviews with Dutch patients. BMJ Open. 2024 Nov 24;14(11):e088201. PMID: 39581732. doi: 10.1136/bmjopen-2024-088201.
25. Joseph AL, Monkman H, MacDonald L, Lai C. Interpreting laboratory results with complementary health information: A human factors perspective. Stud Health Technol Inform. 2024 Jan 25;310:1061–5. PMID: 38269977. doi: 10.3233/SHTI231127.
26. Monkman H, Griffith J, MacDonald L, Joseph AL, Lesselroth B. Why do people use online lab results and what do they look for: A qualitative study. Stud Health Technol Inform. 2022;294:599–603. doi: 10.3233/shti220539.
27. Monkman H, MacDonald L, Nohr C, Tanaka JW, Lesselroth BJ. Hidden in plain sight: Overlooked results and other errors in evaluating online laboratory results. Stud Health Technol Inform. 2022 Jun 6;290:867–71. PMID: 35673142. doi: 10.3233/SHTI220203.
28. Monkman H, Schmit A, Nyholt D, MacDonald L, Lesselroth B. False calm and false alarm: A qualitative study of confusion and misinterpretation of a laboratory results graph. Stud Health Technol Inform. 2025 May 12;326:106–10. PMID: 40357611. doi: 10.3233/SHTI250248.

29. Robinson S, Reed M, Quevillon T, Hirvi E. Patient perceptions and interactions with their web portal-based laboratory results. BMJ Health Care Inform. 2019 Apr;26(1):0. PMID: 31039117. doi: 10.1136/bmjhci-2019-000012.
30. Schultz CL, Alderfer MA. Are on-line patient portals meeting test result preferences of caregivers of children with cancer? A qualitative exploration. Pediatr Blood Cancer. 2018 Nov;65(11):e27306. PMID: 30007016. doi: 10.1002/pbc.27306.
31. Solomon J, Scherer AM, Exe NL, Witteman HO, Fagerlin A, Zikmund-Fisher BJ, editors. Is this good or bad? Redesigning visual displays of medical test results in patient portals to provide context and meaning. 2016 CHI Conference Extended Abstracts on Human Factors in Computing Systems; 2016; San Jose, California, USA: Association for Computing Machinery.
32. Bhalla S, Prasad T, Xie D, Gerber DE. Contemporary trends in reviewing test results through the electronic patient portal among patients with cancer. JAMA Oncol. 2024 Jan 1;10(1):139–40. PMID: 38032648. doi: 10.1001/jamaoncol.2023.5047.
33. Foster B, Krasowski MD. The use of an electronic health record patient portal to access diagnostic test results by emergency patients at an academic medical center: Retrospective study. J Med Internet Res. 2019 Jun 28;21(6):e13791. PMID: 31254335. doi: 10.2196/13791.
34. Krasowski MD, Grieme CV, Cassady B, Dreyer NR, Wanat KA, Hightower M, et al. Variation in results release and patient portal access to diagnostic test results at an academic medical center. J Pathol Inform. 2017 2017/01/01/;8(1):45. PMID: 29226008. doi: 10.4103/jpi.jpi_53_17.
35. McFarland JA, Huang J, Li Y, Gunn AJ, Morgan DE. Patient engagement with online portals and online radiology results. Curr Probl Diagn Radiol. 2023 Mar–Apr;52(2):106–9. PMID: 36030140. doi: 10.1067/j.cpradiol.2022.07.012.
36. Steitz BD, Turer RW, Salmi L, Suresh U, MacDonald S, DesRoches CM, et al. Repeated access to patient portal while awaiting test results and patient-initiated messaging. JAMA Netw Open. 2025 Apr 1;8(4):e254019. PMID: 40198070. doi: 10.1001/jamanetworkopen.2025.4019.
37. Turer RW, Martin KR, Courtney DM, Diercks DB, Chu L, Willett DL, et al. Real-time patient portal use among emergency department patients: An open results study. Appl Clin Inform. 2022 Oct;13(5):1123–30. PMID: 36167337. doi: 10.1055/a-1951-3268.
38. Wood KE, Pham HT, Carter KD, Nepple KG, Blum JM, Krasowski MD. Impact of a switch to immediate release on the patient viewing of diagnostic test results in an online portal at an academic medical center. J Pathol Inform. 2023 2023/01/01/;14:100323. PMID: 37520309. doi: 10.1016/j.jpi.2023.100323.
39. Zhong X, Park J, Liang M, Shi F, Budd PR, Sprague JL, et al. Characteristics of patients using different patient portal functions and the impact on primary care service utilization and appointment adherence: Retrospective observational study. J Med Internet Res. 2020 Feb 25;22(2):e14410. PMID: 32130124. doi: 10.2196/14410.
