# Supplementary material for: Patient Experiences With Online Laboratory Test Presentations From Access to Activation: Systematic Review
Source: J Med Internet Res. 2026 May 29;28:e88259. doi: 10.2196/88259 (PMC13222930; doi:10.2196/88259)
Supplement: Multimedia Appendix 2 [file jmir-v28-e88259-s002.pdf]

Table S1. Detailed Summary of Included Studies

STUDY OVERVIEW (Citation, Study Design & Purpose)	POPULATION AND SAMPLE	Main Variables and Measures	MAIN FINDINGS
**Bhalla, Prasad et al [1]**<br><br>This **retrospective study** examined patterns in patient viewing of laboratory, radiology, and pathology test results through an electronic patient portal before and after the implementation of the 21st Century Cures Act. Data were extracted from EHR and patient portal databases on test result viewing patterns from 2017 to 2022. Time from test result availability to patient viewing was analyzed.	▯ **Country:** United States<br>▯ **Eligibility:** Patients with a cancer diagnosis who received at least one laboratory, radiology, or pathology test result via the Epic MyChart portal<br>▯ **Data sources:** logs of 44,419 patients with 5,570,521 test results released from 2017 to 2022	▯ **Proportion of results viewed:** Percentage of laboratory, radiology, and pathology test results accessed via the portal<br>▯ **Time to review:** Median hours between test availability and patient review<br>▯ **Result type:** Laboratory, radiology, or pathology<br>▯ **Viewer type and timing:** Whether patients or ordering clinicians reviewed results first<br>▯ **Demographic variables:** Race, age, and sex	▯ After the Cures Act, median review time dropped from 77 hours to 6.4 hours.<br>▯ 75% of results were viewed by patients before clinicians in 2022 (increased from 37% in 2017).<br>▯ Black patients were less likely to view results (33%) compared to White (51%) and Asian (58%) patients.<br>▯ Radiology results (64%) and pathology results (64%) were reviewed more frequently than laboratory results (47%).
**Christensen and Sue [2]**<br><br>This **cross-sectional survey** study examined how patients emotionally and behaviorally respond to viewing laboratory test results via an online patient portal. The study also evaluated whether provider communication before patients viewed their results—specifically, conversations that prepared them for what to expect—influenced emotional reactions and follow-up actions.	▯ **Country**: United States<br>▯ **Eligibility criteria**: Adult Kaiser Permanente members who had viewed at least one laboratory test result online in the past 12 months and were part of the Kaiser Member Voice online research panel.<br>▯ **Participant characteristics**: (n=1,546 patients) median age: 58 years; 72% were 50 or older; 56% female; 74% White. Participants were older and more educated than the general portal-user population at KP.	▯ **Emotional reactions**: 10 items assessing satisfaction, appreciation, calm, happiness, relief, worry, confusion, fear, upset, and anger after viewing results.<br>▯ **Follow-up behaviors**: Actions taken after viewing results, such as searching for information online, contacting providers, or discussing results with others.<br>▯ **Physician communication**: Measured whether a provider discussed what to expect prior to test result release.	▯ Participants were older and more educated than the general portal-user population at KP.<br>▯ Over 65% of patients reported feeling satisfied, appreciative, or calm after viewing their lab test results online.<br>▯ Negative emotions were far less common—only 7% reported worry, 6% reported confusion, and fewer than 5% reported feeling afraid, angry, or upset.<br>▯ Patients who had a prior conversation with their doctor about the expected results reported significantly more relief, calm, satisfaction, and less confusion than those who did not (Cohen's d = .15–.26).<br>▯ After viewing their results online, most patients who had prior contact with their doctors, spoke with their family or friends (21%), looked for information on the portal site (20%), made graphs of their results (19%), or looked online for information (18%).<br>▯ Of the patients whose doctors did not set expectations about their results, 60% made

Table 2. Detailed summary of reviewed studies

STUDY OVERVIEW (Citation, Study Design & Purpose)	POPULATION AND SAMPLE	Main Variables and Measures	MAIN FINDINGS
			graphs of their results, 58% looked up information online, 57% looked up information on the portal website, and 55% spoke with their family or friends.
**Foster and Krasowski [3]**<br><br>This **retrospective study** was conducted to examine the use of patient portals (portal activation rates and usage patterns) and rates of accessing diagnostic test results among emergency department patients and to analyze the impact of age, gender, and race. Analysis included 208,635 laboratory tests and 23,504 radiology studies performed at a 60,000-visits-per-year university ED.	**Country:** United States<br>**Eligibility:** Emergency Department (ED) patients who received at least one diagnostic test during their visit between October 2016 and October 2017<br>**Data sources:** logs of 25,361 patients (81.4% White, 8.9% Black, 5% Hispanic/Latino, 1.8% Asian, 3% other; 37.4% had active portal accounts)	**Patient portal activation:** Whether a patient had an active portal account during the study period.<br>**Test viewing rates:** Percentage of laboratory and radiology results accessed by patients through the portal.<br>**Time to access:** Lag time (in days) between test result availability and the time it was viewed by the patient.<br>**Demographic characteristics:** Age group, gender, and self-identified race or ethnicity.	In a retrospective analysis of over 200,000 lab tests and 23,000 radiology studies ordered in a university-based ED, only about 9% of results were viewed by patients through the portal.<br>Portal access was highest among female patients, caregivers of children (0–11 years of age), adults aged 18–60, and individuals identifying as White or Asian.<br>Black and Hispanic/Latino patients were significantly less likely to view their results, even when they had activated portal accounts.<br>Among patients who accessed their results, the median time to viewing after portal release was 2.3 days for lab tests and 3.7 days for radiology reports.<br>View rates were also higher for MRI and infectious disease tests (e.g., blood culture, chlamydia/gonorrhea testing) than for routine panels like electrolytes or blood gases.
**Fraccaro, Vigo et al [4]**<br><br>This **within-subjects controlled experiment** tested whether the design of patient portal displays affects patients' ability to interpret laboratory result risk and select appropriate follow-up actions. Participants reviewed three different test result formats (baseline, contextualized horizontal bars, grouped) in nine clinical scenarios representing low-, medium-, and high-risk results.	**Country:** United Kingdom<br>**Eligibility:** Patients with chronic kidney disease (CKD) who had received a kidney transplant at least 12 months prior<br>**Participants:** 20 kidney transplant patients (20% female, mean age 52 years)	**Accuracy of risk interpretation:** Patients' ability to choose the correct follow-up action for each scenario (e.g., do nothing, schedule an appointment, call the doctor immediately)<br>**Visual search behavior:** Eye-tracking metrics including fixation count, fixation duration, and dwell time during scenario review	Misinterpretation of risk was common—65% of participants underestimated the need for action in at least one scenario, while 70% overestimated the need for action at least once.<br>Participants had the most difficulty interpreting medium-risk results, often failing to select the appropriate follow-up actions.<br>None of the three display formats (baseline, contextualized, grouped) significantly improved patients' ability to accurately interpret risk, particularly in medium-risk scenarios.

Table 2. Detailed summary of reviewed studies

STUDY OVERVIEW (Citation, Study Design & Purpose)	POPULATION AND SAMPLE	Main Variables and Measures	MAIN FINDINGS
Eye-tracking data were also collected to analyze visual search behavior.			- Eye-tracking data showed that participants spent more time viewing contextualized displays, but this did not improve the accuracy of their interpretation of risk.
**Giardina, Modi et al [5]**<br><br>This **qualitative study** explored how patients experience and respond to receiving abnormal test results via a patient portal. Semi-structured telephone interviews were conducted with 13 participants between February and October 2014. Interviews explored participants' test result experiences, information management behaviors, and preferences for receiving sensitive results. Content analysis was used to identify key themes.	- **Country:** United States<br>- **Eligibility:** Adults aged 18+ who accessed a patient portal and received an abnormal test result for themselves or as a caregiver<br>- **Participants:** 13 individuals (11 patients, 2 caregivers), aged 30–80 years, 69% female	- **Portal use behaviors:** How participants used portals to track test results, manage care, and share information with providers<br>- **Test result delivery preferences:** Preferred timing and method of receiving test results (e.g., portal vs. physician contact)<br>- **Emotional responses:** Feelings triggered by receiving abnormal results, such as anxiety, reassurance, or confusion<br>- **Barriers and concerns:** Issues related to portal usability, timing of result release, and clarity of information	- Majority of respondents found that portals to be helpful patient tools.<br>- Participants valued having direct access to test results but reported feeling anxious or confused and having difficulty interpreting results and distress.<br>- Several preferred that their doctor communicate sensitive or life-threatening results directly to them. Some reported a loss of trust or disappointment when that did not happen.<br>- Participants had different notification preferences depending on prior experience and nature of chronic condition.<br>- Patients said the portal lacked adequate context and clarity—some couldn't tell if a provider had even reviewed the result.
**Giardina, Baldwin et al [6]**<br><br>This **mixed-methods study** combined structured and semi-structured interviews to examine patients' emotional and behavioral responses to receiving test results through patient portals.	- **Country:** United States<br>- **Eligibility:** Adults aged 18+ who viewed a test result on a portal between April 2015 and September 2016 at one of four outpatient clinics<br>- **Participants:** 95 patients (mean age = 54.6 years; 65% White; 44% female)	- **Test result understanding and accompanying explanations:** Whether patients received explanatory notes with test results and whether they reported understanding them.<br>- **Emotional responses:** Self-reported emotional reactions, categorized as positive, negative, or neutral.<br>- **Follow-up behaviors:** Actions taken after viewing results (e.g., calling doctors, seeking online information, consulting others).<br>- **Patient Activation Measure (PAM) -** 10-item validated scale	- Most participants reported understanding their test result, with some expressing uncertainty about its health implications—especially when no interpretation was provided in the portal.<br>- About half of participants were told by their physician to check the portal for test results, but nearly two-thirds (63%) did not receive any explanatory note or interpretation from their doctor in the portal.<br>- Participants expressed a desire for clearer explanations, contextual meaning, and guidance on next steps, especially when viewing abnormal results.<br>- Emotional responses were more negative when test results were abnormal, but

Table 2. Detailed summary of reviewed studies

STUDY OVERVIEW (Citation, Study Design & Purpose)	POPULATION AND SAMPLE	Main Variables and Measures	MAIN FINDINGS
		used to assess participants' engagement and capacity for health self-management<br>☐ **Demographic factors:** Age, gender, race, presence of chronic conditions, and general portal usage patterns.	qualitative responses indicated that unmediated release—particularly the absence of explanatory notes—often amplified patients' confusion, concern, or anxiety.<br>☐ Patients with abnormal results were more likely to seek follow-up, including calling their physician (44% vs. 15%) and sending secure messages (33% vs. 19%) compared to those with normal results.
**Hulter, Langendoen et al [7]**<br><br>The study used a **mixed-methods sequential explanatory design** to determine patient preferences for timing of release of different test results (i.e., laboratory, radiology, pathology) via a hospital patient portal and the reasons behind their choices. It involved two phases: Quantitative study: patient portal users were given choices about when they prefer to receive different types of results via the patient portals. Semi-structured interviews were conducted to follow up with seven patients who changed preferences.	☐ **Country**: Netherlands<br>☐ **Eligibility**: Patients using a hospital patient portal with the option to choose delays in test result disclosure.<br>☐ **Participants**: 4592 users of the hospital portal (quantitative phase) and 7 patients who changed their initial preference (interview phase).	☐ **Patient preference for test result timing**: Selected through the portal for each test type (options ranged from 1 to 28 days or "never").<br>☐ **Change in preference**: Number and direction of changes made by users to their initial disclosure timing choices.<br>☐ **Reasons for preference change**: Elicited through semi-structured interviews and analyzed using thematic analysis.<br>☐ **Demographics**: Age and gender of portal users.	☐ Most patients (77%) preferred a 1-day delay for receiving lab results and a 7-day delay for radiology/pathology results.<br>☐ Among the 43 patients who changed their disclosure preferences mid-study, most (78%) shifted toward shorter delays or immediate access, suggesting a growing comfort with online result release.<br>☐ In interviews, participants said they preferred shorter delays because they felt more reassured, it was more transparent, it gave them time to prepare for doctor appointments, and it allowed them to monitor their results and act on them.<br>☐ A few switched to longer delays because the information was hard to understand or lacked details.<br>☐ Preferences were influenced by patients' desire for control and understanding of their health information.
**Hulter, Weggelaar-Jansen et al [8]**<br><br>A **qualitative discourse analysis** using 28 semi-structured interviews was conducted over three years (2018–2021) to explore patients' experiences with being given real-time access to test results via hospital portals and to	☐ **Country:** Netherlands<br>☐ **Eligibility:** Patients aged 16 years or older who had visited outpatient clinics and either used or not used portals for real-time access to test results	☐ **Patient discourses:** Thematic categories reflecting how participants framed their experiences with or expectations of real-time result access, including stress, anxiety reduction, and self-management	☐ Patients described feeling **anxious, confused, or overwhelmed** when viewing unexpected or sensitive results online without prior explanation.<br>☐ Some patients found immediate access reassuring and said it **motivated them to take action** to improve their health or follow up more proactively.

Table 2. Detailed summary of reviewed studies

STUDY OVERVIEW (Citation, Study Design & Purpose)	POPULATION AND SAMPLE	Main Variables and Measures	MAIN FINDINGS
identify advantages, disadvantages, and implications for patient-centered care.	**Participants:** 28 patients (64% female), aged 16–75 years, including 15 with experience and 13 without experience using real-time access portals, recruited from diverse hospitals	**Expectations and values:** Interview themes addressing preferences for clarity, reassurance, or autonomy in accessing lab results<br>**Feature suggestions:** Ideas voiced by participants for improving the patient portal experience (e.g., labels, status indicators, support features)	Many said that immediate access helped them reflect on their health status, **prepare for questions for appointments**, correct any mistakes, participate in decision making and take appropriate follow up actions.<br>Participants emphasized the need for **clear explanations of test results**, including what the numbers meant and what to do next.<br>Some participants recommended adding a **status indicator showing whether the doctor had reviewed the results**, as a way to build trust and reduce worry when results appeared abnormal or unclear.
**Joseph, Monkman et al [9]**<br><br>This **qualitative study** explored how frequent users of lab portals interpret their lab results when complementary health information is presented within the same display. Using a human factors lens, the study investigated comprehension, emotional responses, and usability challenges when viewing potassium level graphs with explanatory text from Testing.com. Data were collected through semi-structured interviews and analyzed using conventional content analysis.	**Country**: Canada<br>**Eligibility:** English-speaking adults, frequent users of online lab portals<br>**Participants:** 24 participants (95.8% female; age 24–64 years; most had post-secondary education)	**Perceived benefits:** Participant comments reflecting appreciation for having contextual health information directly next to test results.<br>**Information overload:** Reactions to the volume and complexity of text shown alongside lab graphs.<br>**Misinterpretation:** Incorrect assumptions or conclusions drawn from the information presented (e.g., believing high potassium levels warrant more intake).<br>**Confusion:** Difficulty understanding the information or knowing what actions to take.	This study found 4 emerging themes:<br>**Benefits of Collocated Information**: Participants valued having high-quality health information alongside lab results.<br>**Information Overload**: Participants felt overwhelmed by the amount of information provided, which could lead to cognitive overload and difficulty in processing the data.<br>**Misinterpretation**: Some participants misinterpreted the complementary information, leading to incorrect assumptions about their health.<br>**Confusion**: Participants did not understand the additional information or confused about what actions to take.
**Krasowski, Grieme et al [10]**<br><br>This **retrospective study** examined patterns in patient access to diagnostic test results (clinical laboratory, anatomic pathology, and radiology) released via patient portals. The study focused on variations in patient viewing behavior	**Country:** United States<br>**Eligibility:** Patients who underwent diagnostic tests at an academic medical center over six months (January–June 2016)	**Release method:** Manual (provider-triggered) vs. automatic release (auto-release after 1 or 4 business days).<br>**Patient access rate:** Percentage of test results viewed within defined time frames (within 2	Across inpatient and emergency settings, test result viewing was uniformly low (<10%). In the outpatient setting, viewing rates were higher (~30% overall).<br>Manual release was more common for outpatient tests, while inpatient and

Table 2. Detailed summary of reviewed studies

STUDY OVERVIEW (Citation, Study Design & Purpose)	POPULATION AND SAMPLE	Main Variables and Measures	MAIN FINDINGS
based on result release method (manual vs. automatic), type of clinical encounter (outpatient, inpatient, emergency), and patient demographics (age, gender). Electronic health record data from a 6-month period were analyzed descriptively.	**Participants:** 59,388 patients across outpatient clinics, inpatient units, and emergency department	hours, 8 hours, or over 7 days after portal release).<br>**Demographic factors:** Age, gender, encounter type (outpatient, inpatient, emergency department).<br>**Portal activation rate:** Percentage of patients who had activated patient portal accounts.	emergency department tests were predominantly auto released.<br>Manual result releases were viewed more often than auto-released results.<br>Approximately 20% of outpatient test results were viewed by patients within 8 hours of release to the portal.<br>Females and individuals aged 20-45 accessed their test results more frequently than males and older adults. Children aged 12-17 had very low access rates, likely due to institutional policies restricting parental proxy access.
**Lustria, Aliche et al [11]**<br><br>A **cross-sectional web-based survey** was conducted to explore patients' use of patient portals to view lab test results and factors influencing lab test comprehension.	**Country:** United States<br>**Eligibility:** Adults aged 18 or older, English-speaking, with one or more chronic conditions<br>**Participants:** 276 adults (mean age = 50.7 years, SD = 15.5; 72.5% White; 55.4% female)	**Lab test comprehension**: 19-item comprehension test assessing participants' ability to identify lab results outside normal reference ranges for lipid, metabolic, and hemoglobin A1c panels (custom measure developed for this study).<br>**Numeracy**: 3-item Subjective Numeracy Scale (McNaughton et al., 2015), 6-point Likert scale (e.g., "How good are you at working with fractions?").<br>**eHealth literacy**: 8-item eHealth Literacy Scale (eHEALS; Norman & Skinner, 2006), 5-point Likert scale (e.g., "I know how to use the Internet to answer my health questions.").<br>**Portal use**: Frequency and preference for accessing lab results through patient portals versus traditional methods (custom measure).	Participants who were White, lacked a college degree, and had higher eHealth literacy were more likely to use the portal to view results.<br>Patients who were older, had fewer chronic conditions, and who used patient portals to view lab test results had significantly higher lab test comprehension scores.<br>Despite higher comprehension scores, older adults and those with multiple chronic conditions were more likely to say they struggled with understanding test results displayed in the portal.<br>Many participants—especially older adults—reported difficulty interpreting lab values, often due to unclear medical terminology or unfamiliar reference ranges.<br>Over half of participants preferred in-person or provider-led explanation, citing confusion or difficulty interpreting values independently.<br>Although 60.4% of participants reported accessing lab test results through a patient portal, most still preferred to have a physician explain abnormal results.

Table 2. Detailed summary of reviewed studies

STUDY OVERVIEW (Citation, Study Design & Purpose)	POPULATION AND SAMPLE	Main Variables and Measures	MAIN FINDINGS
		• **Barriers to portal use**: Checklist of reasons for not using portals, such as preference for doctor's explanation, uncertainty on interpreting results (custom measure).<br>• **Predictors of comprehension**: Age, race, education level, eHealth literacy, numeracy, number of chronic conditions, and portal use (analyzed using logistic regression).	• Roughly half of participants—particularly those aged 18–34—said they only wanted to know results if they were abnormal and tended to disregard values that were within the normal range.
**Mak, Smith Fowler et al [12]**<br><br>This **retrospective, cross-sectional survey** examined whether accessing lab test results online affects patients' experiences with turnaround time, comprehension, and anxiety. The researchers compared two groups: individuals who accessed results through a web-based portal (service users) and individuals who received results via traditional methods (comparison group).	• **Country:** Canada (British Columbia)<br>• **Eligibility:** Adults who had laboratory tests within the past 12 months and who had accessed their results in-person, online, via mail, email, or over the phone.<br>• **Participants:**<br>Service Users (n = 2047): 70.3% female; age groups – 18–34 (9.75%), 35–54 (28.6%), 55+ (61.65%).<br>Comparison Group (n = 1245): Balanced by age and gender; excluded those who accessed results online; 83.8% female.	• **Wait time:** Measured by self-reported time from testing to result receipt, categorized as "within a few days," "within a week," or "longer."<br>• **Comprehension of lab test results:** Measured using a self-rated 10-point scale of confidence in understanding results, and a yes/no question on whether the individual understood if follow-up was needed.<br>• **Anxiety:** Measured using the Global Anxiety-Visual Analog Scale (GA-VAS), a 100-mm line from "no anxiety" to "worst imaginable anxiety" (Kindler et al., 2000).	• Although portal users had faster access, only 76% reported high confidence in comprehending their test results, compared to 85% of non-users.<br>• Younger, less educated, and those who first learned about their tests online were significantly more likely to have trouble understanding their test results.<br>• Among patients with chronic conditions, portal users reported significantly less anxiety about test results than non-users.<br>• Users who were unclear about the need for follow-up were more likely to report higher anxiety, suggesting that comprehension and clarity affect emotional responses.
**McFarland, Huang et al [13]**<br><br>This **retrospective study** examined patient engagement with online portals, comparing usage patterns for laboratory results and radiology reports. Researchers analyzed EMR data from March 2017 through March 2018 to	• **Country:** United States<br>• **Eligibility:** Patients of a single academic tertiary care center with available EMR data from March 2017 to March 2018<br>• **Data sources:** user logs of (N = 424,422; 138,783 were	• **Portal enrollment:** Coded as a binary variable based on whether the patient had enrolled in the portal.<br>• **Demographics:** Age, gender, race, income, education level, and insurance status were	• Among all patients in the health system, 32.7% were enrolled in the patient portal during the 13-month study period.<br>• Of those who enrolled, 47.2% accessed lab results, while only 27.1% accessed radiology results, based on clicks within the respective portal tabs.

Table 2. Detailed summary of reviewed studies

STUDY OVERVIEW (Citation, Study Design & Purpose)	POPULATION AND SAMPLE	Main Variables and Measures	MAIN FINDINGS
identify predictors of portal enrollment and interaction with the "Laboratory" (LABTAB) and "Radiology" (RADTAB) tabs.	enrolled in the patient portal). Median age = 49 years; 58% female; 58.5% White; 30.2% Black.	extracted to assess predictors of portal use.<br>- **Portal usage:** Measured by tracking interactions with the LABTAB (laboratory results) and RADTAB (radiology results) tabs.	- Portal users were significantly more likely to be older, female, and White, with higher levels of education, higher household income, and more frequent outpatient visits; these predictors remained significant in multivariable models.
**Monkman, Griffith et al [14]**<br><br>This **qualitative study** explored why patients access online lab results and what information they seek when using patient portals. Participants completed a survey and participated in semi-structured interviews via Zoom. Transcripts were analyzed using affinity diagramming to identify common motivations and search behaviors related to lab result use.	- **Country**: Canada<br>- **Eligibility criteria:** Adults aged ≥19 years with at least two years of experience using online lab results portals.<br>- **Participants:** N=25; ages 18–74 years; 80% born in Canada; 92% with post-secondary education; 72% with one or more chronic conditions; all English-speaking.	- **Motivations for use:** Interview responses categorized into six themes reflecting users' reasons for accessing lab results (e.g., reassurance, health learning, treatment tracking).<br>- **Information sought:** Interview responses were categorized into two themes: identifying normal/abnormal values and monitoring trends over time.<br>- **Portal familiarity:** Participants reported duration and frequency of portal use as part of a demographic survey (≥2 years of use required; 24 accessed results several times a year or more).	- Participants described multiple motivations for viewing their lab results online, including tracking chronic conditions, monitoring treatment effectiveness, obtaining reassurance, and increasing their understanding of test results.<br>- Many valued rapid access, describing portals as a tool to improve patient safety by catching abnormal results early or avoiding missed communications.<br>- Participants routinely scanned for abnormal or normal values and checked trends over time to make informed decisions about self-care or whether to follow up with a doctor.<br>- Viewing results was seen as a way to stay informed between appointments and to prepare for future consultations.
**Monkman, MacDonald et al [15]**<br><br>This study employed **qualitative research** design to examine how individuals without medical expertise interpret abnormal laboratory results in mock online lab test portals, identify errors, and evaluate potential causes of these errors and their implications for the design of consumer-facing lab results. Participants were presented with a **mock online laboratory test report** and were asked to identify if there were any values outside the reference ranges.	- **Country**: Canada<br>- **Eligibility:** Adults aged ≥19 years with prior experience accessing lab results online; non-clinicians<br>- **Participants:** 25 participants (92% female); 80% born in Canada; all English-speaking; most had post-secondary education	- **Abnormal result detection:** Participants were asked to identify results outside the reference range; detection accuracy was evaluated across four flagged results and one unflagged abnormal value (eGFR).<br>- **Misidentification of normal values:** Incorrectly identifying a normal result as abnormal.<br>- **Error type analysis:** Frequency of missed flagged results, overlooked unflagged results, and false positives were recorded.	- Most participants made at least one error identifying abnormal results in a mock lab report—commonly overlooking flagged values that lacked clear reference ranges or visual cues.<br>- Only 5 out of 13 participants correctly identified the low eGFR value as abnormal; several falsely identified normal results as problematic, indicating challenges in recognizing clinically significant values.<br>- Participants struggled with unfamiliar medical terms, inconsistent or hidden reference ranges, and unclear explanations. Some were unsure if they had diabetes, kidney disease, or

Table 2. Detailed summary of reviewed studies

STUDY OVERVIEW (Citation, Study Design & Purpose)	POPULATION AND SAMPLE	Main Variables and Measures	MAIN FINDINGS
		**User feedback:** Qualitative comments were analyzed to identify barriers to interpretation related to visual design, layout, and information presentation.	other conditions, reflecting confusion about test meaning.<br>Participants expressed frustration with lengthy reports, dense formatting, and technical language. Many said the layout contributed to cognitive overload and difficulty extracting key information.<br>Several participants requested simpler formats with visual flags, color cues, or icons to highlight abnormal values and urgency more clearly.
**Monkman, Griffith et al [16]**<br>This **web-based survey** study investigated barriers and facilitators to using online laboratory result portals, focusing on usability, clarity of information, and user preferences for result display features. Closed-ended survey items were analyzed descriptively, and open-ended responses were thematically grouped using affinity diagramming.	**Country**: Canada<br>**Eligibility**: Adults aged ≥19 years with experience using at least one laboratory results portal; not healthcare professionals or trainees<br>**Participants**: Survey participants (n = 30); 87% female; 57% age ≥45; 77% born in Canada; 73% reported at least one chronic condition	**Portal use history:** Duration and frequency of use<br>**Perceived usability and clarity:** Ratings of ease of navigation, display formatting, and information availability<br>**Display preferences:** Use of features like color-coding, trend graphs, and visual formatting<br>**Comprehension barriers:** Difficulty understanding terminology and result meaning<br>**Net Promoter Score (NPS):** Likelihood of recommending the portal (0–10 scale)	Participants described a variety of positive experiences, including feeling informed, independent, and empowered by having direct access to lab results online. Several noted this helped them better manage chronic conditions or prepare for clinical visits.<br>Despite overall usability ratings being moderate to high, many reported confusion and frustration, especially with understanding medical terminology, test acronyms, and vague result descriptions. Several participants noted they still turned to the internet to interpret results.<br>Participants identified comprehension barriers, including difficulty interpreting out-of-range values, understanding test purposes, and distinguishing clinical relevance. These issues were compounded by missing contextual explanations or links to educational content.<br>Many expressed a need for clearer explanations, including plain language descriptions, definitions of medical abbreviations, and contextual information about normal vs. abnormal values and what actions might be needed.

Table 2. Detailed summary of reviewed studies

STUDY OVERVIEW (Citation, Study Design & Purpose)	POPULATION AND SAMPLE	Main Variables and Measures	MAIN FINDINGS
			Participants favored visual enhancements, such as bolding or color coding for abnormal values, alternating row shading for readability, and improved spacing, layout, and font size to make results easier to scan.<br>Several participants suggested adding follow-up support features, such as automated notifications when results were available and links to additional resources or options to message providers.
**Monkman, MacDonald et al [17]**<br><br>**Mixed-methods user study** that examined health consumers' preferences for four online laboratory result display formats (Tabular, Annotated, Visual, Trends + contextual information) using portal-like lab result stimuli.	**Country**: Canada<br>**Eligibility**: Adults with prior experience viewing online lab results<br>**Participants**: N = 24; age range 19–74 years; all English-speaking; all at least high school educated; most reported at least one chronic condition	**Display format:** Four lab result display types (Tabular, Annotated tabular, Visual summary with color-coded out-of-range values and suggested actions, Trends + contextual information).<br>**Display preference:** Ranking of each display from favorite (1) to least favorite (4), analyzed across formats.<br>**Perceived clarity and usefulness:** Participants stated reasons for ranking each format, including aspects that helped or hindered understanding of lab results.	Fifteen of 24 participants ranked the Visual display as their favorite and 11 ranked the Tabular display as their least favorite; only this pair differed significantly in overall rankings ($\chi^2(3) = 10.8$, $p = .013$; Visual vs. Tabular $Z = -2.746$, $p = .006$).<br>Participants reported that the traditional Tabular display was difficult to interpret without medical knowledge, whereas the Visual display's color indicators and summary of out-of-range values made it easier to see which results might need attention.<br>Several participants appreciated displays that included brief explanations and trend information, and many said their ideal interface would combine color-coded summaries with trends or annotated explanations, with some wanting the option to switch between visual and tabular formats.
**Monkman, Schmit et al [18]**<br><br>**Qualitative think-aloud study** that examined how adults interpret a longitudinal potassium laboratory results graph modeled on a community lab portal, focusing on sources of	**Country**: Canada<br>**Eligibility:** Adults with prior experience viewing online lab results<br>**Participants:** N = 24; age range 19–74 years, all English-speaking, all at least high school educated;	**Graph stimulus**: Longitudinal potassium graph with two values (4.0 and 5.3 mmol/L) over 11 days and a reference range of 3.5–5.2 mmol/L, where the second value was slightly above the upper limit.<br>**Confusion and misinterpretation**: Content-analysis codes indicating when	Eighteen of 24 participants showed confusion about at least one of nine graph features (e.g., Y-axis, X-axis, reference range, date range, line, "change importance"), and 12 misinterpreted at least one of six elements (e.g., test frequency, meaning of the change, flags, line, data point).<br>Only two participants stated they would urgently follow up with a healthcare provider

Table 2. Detailed summary of reviewed studies

STUDY OVERVIEW (Citation, Study Design & Purpose)	POPULATION AND SAMPLE	Main Variables and Measures	MAIN FINDINGS
confusion, misinterpretation, and intended follow-up actions.	nearly all reported reviewing their lab results online a few times per year or more.	participants were confused by or misinterpreted elements such as axes, reference range, date range, line representation, or perceived importance of the change.<br>- **Intended follow-up**: Self-reported actions if the results were their own (urgent contact with a provider, eventual follow-up, searching online, or not in a hurry)	about the elevated potassium value; four said they would use Google, eight would follow up eventually, and 12 reported not being in a hurry to seek care.<br>- The authors concluded that participants' difficulties with basic graph components and the low rate of urgent follow-up in the presence of an out-of-range value indicate a risk of underestimating clinically important changes when lab results are presented solely as graphs.
**Morrow, Azevedo et al [19]**<br><br>This **experiment** examined whether different patient portal message formats (standard numerical table, plain-language explanation, graphically enhanced display, and video-enhanced format) influence older adults' understanding and response to cholesterol and diabetes test results. Participants were randomly assigned to one of four formats and reviewed results for fictitious patients at low, borderline, or high risk, followed by structured questionnaire-based assessments of memory, perceptions, and behavioral intentions.	- **Country**: United States<br>- **Eligibility**: Adults aged 60 and older; community-dwelling, English-speaking; no cognitive or sensory impairments<br>- **Participants**: 144 older adults (mean age = 71.9 years); 71.5% female	- **Memory and Interpretation**: Verbatim memory (recall of numerical values) and gist memory (interpretation of risk meaning)<br>- **Risk perception**: Likelihood of developing complications (Garcia-Retamero & Cokely, 2011)<br>- **Affective response**: Positive/negative emotional ratings (9-point scale)<br>- **Attitude** toward taking cholesterol-lowering medication<br>- **Intention to act**: Likelihood of taking medications, exercising, or making changes to diet<br>- **Message satisfaction**: Usefulness rating of test result format	- Verbally and video-enhanced formats improved gist memory compared to the graphically enhanced format, while verbatim memory did not differ across formats.<br>- The graphically enhanced format led some participants to overestimate risk in low- and borderline-risk scenarios.<br>- Affective responses became more negative as risk level increased, regardless of display format.<br>- Presentation format influenced attitudes toward and intentions to take medication, particularly for lower-risk results, but had limited effects on intentions to change diet or exercise.
**Nystrom, Singh et al [20]**<br><br>This study used a **mixed-methods, user-centered design approach** to develop and evaluate a patient-facing prototype for reviewing lab test results via a portal. Drawing from prior research on patient information needs, the	- **Country**: United States<br>- **Eligibility**: Adults 18+ years old<br>- **Participants** (N=14): patients aged 25–73 years (mean age = 43 years), 71%	- **Usability:** 10-item System Usability Scale (SUS) with 5-point response options (Brooke, 1996; e.g., "I thought the system was easy to use")<br>- **User feedback:** Post-session questionnaire on satisfaction,	- Early interface versions revealed key comprehension challenges, such as patient confusion over how HDL values were interpreted compared to other cholesterol metrics (e.g., why higher values for HDL were better).

Table 2. Detailed summary of reviewed studies

STUDY OVERVIEW (Citation, Study Design & Purpose)	POPULATION AND SAMPLE	Main Variables and Measures	MAIN FINDINGS
authors created an interactive prototype and iteratively refined it across six design cycles. Evaluation involved multidisciplinary feedback, expert reviews, and usability testing with patients using think-aloud protocols, the System Usability Scale (SUS), and post-session questionnaires.	had prior work experience in healthcare.	information needs, and next-step intentions<br>**Comprehension support:** Think-aloud data identifying usability issues and sensemaking challenges<br>**Task performance:** Ability to interpret lipid profile, hepatitis B, and liver function test results within the interface	After viewing their results, 64% said they would consider making lifestyle changes, while 35% indicated they would follow up with their physician to discuss interpretations and next steps.<br>In earlier versions of the prototype, over a third (36%) of participants said they would turn to the internet to better understand their results, particularly seeking explanations for clinical terms and implications. Over successive versions, users became less reliant on external internet searches and more confident in interpreting results directly from the interface.<br>Some also wanted personalized benchmarks, such as average values for people like themselves.
**Pillemer, Price et al [21]**<br><br>This **mixed-methods study** evaluated how direct release of lab test results via a patient portal influenced patient engagement, safety, anxiety, and healthcare utilization. Quantitative components included analysis of portal log data, EHR records, and a survey of 6,368 active portal users. Qualitative insights came from interviews with 13 patients and physicians serving on an EHR advisory panel.	**Country**: United States<br>**Data sources:** logs of 14,441 patient portal users during a one-year period (4/1/2011 to 3/31/2012) were collected from UPMC Health System, mean age 51.8 years; 45.2% female; 91.4% White.<br>**Participants**: Survey respondents (n=6,368) were active portal users. A separate subset of 13 patients (mean age not reported) participated in qualitative interviews. Additionally, EHR advisory panel physicians provided perspectives via interviews.	**Test result release mode**: Physician-released vs. auto-released results<br>**Portal engagement**: Frequency of logins, percentage of sessions with test result views, proportion of users who viewed results<br>**Patient experience**: Perceived usefulness, anxiety, provider accessibility, preparedness, ownership<br>**Healthcare utilization**: Number of office visits and phone calls following result release	Viewing auto-released test results—especially normal results without provider interpretation—was associated with a statistically significant increase in follow-up contact. Office visits rose by 3.9% and phone calls by 4.9% ($p < .001$). This pattern was not observed for manually released results.<br>Patients used early access to test results to research information, track values like HbA1c, and prepare questions in advance, reflecting greater readiness and engagement—even when results were not explained by a clinician.<br>Some patients experienced anxiety after viewing abnormal results without interpretation, especially when results were released on weekends or contained unexplained flags.<br>Providers emphasized that brief comments such as *"nothing concerning here"* could

Table 2. Detailed summary of reviewed studies

STUDY OVERVIEW (Citation, Study Design & Purpose)	POPULATION AND SAMPLE	Main Variables and Measures	MAIN FINDINGS
			reduce anxiety and prevent unnecessary follow-up.
**Robinson, Reed et al [22]**<br><br>This **qualitative, cross-sectional study** aimed to understand why patients use an electronic patient portal (myCARE) to access laboratory results and how this access affects their comprehension, engagement, and care behaviors. Researchers conducted in-person, semi-structured interviews with 21 adult myCARE users recruited from a single primary-care practice; interviews focused on usage patterns, understanding of results, emotional reactions, and perceived benefits or drawbacks. Data were analyzed using grounded theory—open, axial, and selective coding performed concurrently with data collection until thematic saturation was reached.	**Country**: Canada.<br>**Eligibility**: Adult patients of one primary care physician who actively used the lab-results feature of the "myCARE" portal.<br>**Participants** (N=21): 42.9% female, average age 60-69 years old	**Portal engagement**: reasons for sign-up, frequency of use, and test types viewed.<br>**Usability**: ease of use, navigation challenges, and suggestions for improvement.<br>**Comprehension**: understanding of medical terms and reference ranges; strategies used for clarification (e.g., web searches, messaging providers).<br>**Emotional response**: feelings of reassurance or anxiety after viewing results.<br>**Contextual support**: presence and value of physician comments on results.<br>**Healthcare impact**: effects on visit preparation, provider communication, and health behaviors (e.g., lifestyle changes).	Viewing results empowered patients to monitor their health and engage in health-related behaviors, with some making lifestyle changes based on the results.<br>Patients felt that portal access reduced unnecessary appointments, saved time, freed up clinical resources for others, and made it easier to address minor concerns without a visit.<br>Some experienced distress when receiving alarming results without explanation and preferred in-person delivery for concerning findings.<br>To understand test results, they reported relying on internet searches, family members, or follow-up discussions with their physician to make sense of unfamiliar terms.<br>Patients wanted reassurance that their physician had seen their results and appreciated features such as annotations, alerts, and the ability to confirm that results had been reviewed.<br>Participants wanted clearer layouts, more visuals, and broader access to related data (e.g., test explanations) to improve usability.
**Scherer, Witteman et al [23]**<br><br>This study employed a **web-based, between-subjects experimental study** to examine the impact of including clinically appropriate goal ranges outside the standard range in visual displays of laboratory test results. Participants were randomly assigned to view hypothetical glycated hemoglobin	**Country**: United States.<br>**Eligibility**: Participants were U.S. adults aged 18 or older, recruited via Survey Sampling International's (SSI) national online panel. The sample included individuals with and without type 2 diabetes, and quotas were used to ensure demographic diversity by	**Comprehension**: Measured by participants' ability to identify their test results relative to the goal range and their expectations for future results.<br>**Negative reactions**: Assessed via discouragement and urgency to contact a doctor (using Likert-type scales).	Replacing the standard range with a clinically appropriate goal range improved comprehension and reduced negative emotional responses, especially when test results were near but not far outside the goal.<br>Including multiple ranges (e.g. both a standard and a goal range) on the same visual was confusing and less effective.<br>Participants who viewed the block-style number line with color-coded categories had

Table 2. Detailed summary of reviewed studies

STUDY OVERVIEW (Citation, Study Design & Purpose)	POPULATION AND SAMPLE	Main Variables and Measures	MAIN FINDINGS
(HbA1c) results using three different formats:<br>- a table<br>- a simple two-colored number line<br>- a number line with diagnostic categories indicated via colored blocks.	age, gender, education, race/ethnicity, and health condition.<br>- **Participants** (N=6,766): recruited via a nationwide web-based panel, average age 49.1 years, 50.9% female, 38.8% with type 2 diabetes.		lower comprehension and felt more discouraged compared to those who saw simpler formats (e.g., table or two-tone line).<br>- Participants with diabetes were less likely to correctly interpret moderately abnormal results than participants without diabetes, but they also felt less discouraged and less urgency to contact a provider.<br>- Across display types, urgency to contact a doctor was lower when goal ranges were presented alone, suggesting that reference framing affected how seriously patients perceived the result.
**Schultz and Alderfer [24]**<br>This **qualitative study** used semi-structured interviews to explore caregivers' preferences for receiving pediatric cancer test results and their experiences using online patient portals. Interviews were conducted with caregivers of children with cancer at a single pediatric institution. Transcripts were analyzed using inductive qualitative content analysis to identify themes related to result communication preferences, perceived benefits and drawbacks of portal access, and the emotional context surrounding information delivery.	- **Country**: United States.<br>- **Eligibility**: Caregivers of children with cancer from a mid-Atlantic children's hospital.<br>- **Participants** (N=19): 18 parents and 1 grandparent; 78.9% women; 73.7% White; average age 40.4 years.	- **Delivery preference:** Whether caregivers prioritized fast result delivery, preferred a specific communication mode (e.g., in person or phone), or weighed both factors equally.<br>- **Anxiety influence:** How caregiver anxiety shaped communication preferences and portal use.<br>- **Portal engagement:** Sign-up and usage status; accessed features such as test result viewing, appointment management, and messaging.<br>- **Perceived portal pros/cons:** Benefits (e.g., speed, longitudinal tracking) vs. drawbacks (e.g., confusing terminology, early result release).	- Caregivers preferred speed of delivery over mode of communication, with most preferring notification by their provider either via phone or in person, and only supplemented by electronic means (e.g., via portal or email).<br>- Majority of caregivers preferred to receive abnormal or concerning results directly from their doctor although this changed over time as they became more familiar with their child's condition.<br>- To ease anxiety, many caregivers said they wanted to view their test results as soon as possible, even if they usually preferred hearing about their results from a provider directly.<br>- Some participants noted that portal access could reduce burden on clinic staff by decreasing routine calls about lab results, allowing providers to focus on higher-acuity care.<br>- Portal features that allowed caregivers to track trends, visualize data, and store records were seen as helpful, especially when managing a child's long-term treatment.

Table 2. Detailed summary of reviewed studies

STUDY OVERVIEW (Citation, Study Design & Purpose)	POPULATION AND SAMPLE	Main Variables and Measures	MAIN FINDINGS
**Solomon, Scherer et al [25]**<br><br>This **user-centered design study** explored how to improve patient comprehension and interpretation of lab results presented via patient portals. Through an iterative design process, the researchers developed multiple prototype visualizations incorporating contextual elements—such as personalized goals, harm thresholds, and standard ranges. Participants completed in-person think-aloud interviews across three design waves. Researchers gathered qualitative feedback on understanding, emotional reactions, clarity of information sources, and desired next steps.	**Country**: United States<br>**Eligibility**: Individuals with type 1 or type 2 diabetes, or friends/family members of people with diabetes<br>**Participants** (N = 18): Median age range 50–59 years; 47% female	**Design Features:**<br><br>**Contextual indicators:** Standard reference ranges, harm thresholds, and patient-specific goals<br>**Visual cues:** Color coding (e.g., red-amber-green schemes), graphical outlier annotations<br>**Information density:** Minimalist vs. text-heavy explanations<br>Participant Feedback Themes:<br>**Comprehension:** Ease of identifying normal vs. concerning values at a glance<br>**Emotional response:** Feelings of anxiety or reassurance triggered by color and framing<br>**Clarity of attribution:** Differentiating whether values were set by physicians vs. general population standards<br>**Support needs:** Requests for follow-up actions, explanations, or links to more information	Color improved comprehension and risk interpretation (red for abnormal results) but also triggered different emotional responses— triggering appropriate action or causing unnecessary anxiety.<br>Participants wanted simple, minimalist graphs for easy interpretation, but also requested more information, larger fonts, and better readability. Limited screen space—especially on mobile—poses a major design challenge.<br>Adding contextual information to test results often sparked users' desire for even more detailed information or physician guidance, highlighting a design tension: portals can't deliver all the nuance patients want and can also cause clutter and distraction.<br>Participants expressed the need for clearer attribution of information sources (e.g., doctor vs. general guidelines) to enable them to assess relevance of credibility.<br>Participants wanted visual cues reflecting their doctor's evaluation of abnormal test results and the ability to contact their doctor immediately when results seemed concerning.
**Steitz, Turer et al [26]**<br><br>This **large, multisite survey** study examined how patients and caregivers perceive the immediate release of test results through online patient portals, including its impact on anxiety, engagement, and overall preferences. Surveys were distributed via email to individuals who accessed test results through a portal over a one-year period.	**Country:** United States<br>**Eligibility:** Patients and caregivers who accessed test results through a patient portal between April 2021 and April 2022<br>**Participants (N = 8,139):** Median age 64 years; 63% female; 84.8% White	Survey instrument adapted from Turer et al. (2021), originally designed to assess patient reactions to immediate COVID-19 test result release:<br>**Perceived result normality:** Patient-perceived normal vs. not normal<br>**Level of worry:** Ordinal scale from "never worried" to "much more worried"	Nearly all respondents (96%) said they preferred receiving test results through the patient portal as soon as they were available—even before a provider had reviewed them.<br>Of participants who viewed their test results, majority (87.4%) were contacted by their provider, most commonly through the patient portal (61%).<br>Online access generally reduced or had no effect on patient anxiety for most users where

Table 2. Detailed summary of reviewed studies

STUDY OVERVIEW (Citation, Study Design & Purpose)	POPULATION AND SAMPLE	Main Variables and Measures	MAIN FINDINGS
		**Pre-counseling:** Whether the patient received advance communication from a clinician (yes/no)<br>**Information-seeking behavior:** Whether patients looked for more information after viewing results<br>Preferred result delivery method: Portal, phone, or mail<br>Demographics and test characteristics	nearly half felt less worried even when seeing them before hearing from a provider.<br>Most patients with abnormal results (84%) said their level of worry either stayed the same or decreased after accessing results online, suggesting that even when results were not normal, online access generally did not heighten anxiety for most users.<br>Many respondents (39.9%) sought additional information through internet searches after viewing results.
**Steitz, Turer et al [27]**<br><br>This **retrospective observational study** used EHR and portal log data to analyze patient behavior while awaiting lab test results through a patient portal. The study aimed to explore how result release timing and test sensitivity influence two key engagement behaviors: refreshing the portal and messaging providers. By linking these behaviors to test sensitivity levels (i.e., whether results are considered emotionally sensitive or routine), the study provides insights into how patients interact with portal systems before and after result availability.	**Country**: United States<br>**Eligibility**: Adult patients at Vanderbilt University Medical Center with outpatient lab tests and active patient portal accounts<br>**Data sources**: logs of 329,317 active portal users (for ~1.2 million tests released).	Result sensitivity: Tests were classified as high-sensitivity (e.g., biopsy or cancer screening) or low-sensitivity (e.g., cholesterol, routine metabolic panels), based on clinical urgency.<br>Refreshing behavior: Log data indicated whether patients refreshed the lab results page before the result was posted.<br>Post-result messaging: Whether the patient sent a secure message to their provider within 6 hours after the result was released.<br>Patient characteristics: Age, sex, test type	Over one-third of patients refreshed the portal while waiting for results, and refresh behavior was more common for results of high-sensitivity tests (e.g., biopsy or cancer screening).<br>Frequent refreshing behavior was more common among patients who were female, younger, White, non-Hispanic, and English-speaking. However, compared to privately insured participants, Medicare patients had higher odds of refreshing high-sensitivity results.<br>Patients who refreshed the portal more frequently were also more likely to message their clinician via the portal within 24 hours of viewing low-sensitivity results (e.g., routine blood tests) compared to high-sensitivity results.<br>Patients who enabled result notifications or had more same-day test orders were more likely to check the portal repeatedly before results were released.
**Steitz, Guide et al [28]**<br><br>This **quality improvement study** using an interrupted time series design evaluated whether releasing laboratory	**Country**: United States<br>**Eligibility**: Adult outpatients (≥18 years) who received laboratory test results via	**Patient-initiated messaging rate**: Proportion of reviewed results followed by a patient-initiated message within 24 hours	Introducing patient-friendly educational materials was not associated with a clinically meaningful overall change in patient-initiated

Table 2. Detailed summary of reviewed studies

STUDY OVERVIEW (Citation, Study Design & Purpose)	POPULATION AND SAMPLE	Main Variables and Measures	MAIN FINDINGS
test results in a patient-friendly educational format through a patient portal was associated with a reduction in patient-initiated messaging. Educational materials — including a plain-language test description, basic interpretation with a graphical display of the result and reference range, and a note indicating clinician follow-up if needed — were integrated into the Epic MyChart portal at Vanderbilt University Medical Center on August 7, 2024, for the test types associated with the highest message volumes. Portal audit log data were extracted for all adult outpatient results released between January 1 and December 31, 2024.	the VUMC MyChart portal during 2024.<br>- **Participant characteristics**: 205,139 patients who reviewed 829,902 results; mean age 51.0 years (SD 17.8); 63.5% female; 79.1% White; 10.6% Black; 5.1% Hispanic; 59.6% commercially insured.	of result release; derived from portal audit log timestamps.<br>- **Weekly message volume**: Absolute number of patient-initiated messages per week.<br>- **Order setting**: Whether result was ordered in primary care or specialty care.<br>- **Result status**: Whether result was flagged as abnormal or normal.<br>- **Demographic and contextual factors**: Age group, sex, race, ethnicity, preferred language, insurance type, time enrolled in portal, and whether the patient reviewed the result before the ordering clinician.	messaging (17.5% pre-intervention vs. 17.3% post-intervention; p = .22).<br>- A statistically significant reduction in messaging was observed for tests ordered in primary care (AME −0.8%; 95% CI −1.2% to −0.5%; p < .001) but not for tests ordered in specialty care.<br>- Among all reviewed results, 69.4% were viewed by patients before their ordering clinician.<br>- Patients who did not review results still initiated messages at a rate of 6.8%, suggesting that messaging occurs for reasons beyond direct result review.
**Struikman, Bol et al [29]**<br><br>This web-based pre-post experiment examined whether the design of patient portals for viewing blood test results influences patient health engagement (PHE), and whether this effect differs based on the test outcome (normal, partially abnormal, or all abnormal). A 2×3 between-subjects design exposed participants to fictional blood test results using either a basic portal or one enhanced with visual aids and explanatory text. PHE was measured before and after exposure to assess how the presentation style and result type influenced cognitive, emotional, and behavioral aspects of engagement.	- **Country:** Netherlands<br>- **Eligibility:** adults 18+ years recruited from the Nivel Dutch Health Care Consumer Panel<br>- **Participants (N = 487):** Average age 52.8 years; 50.3% female	- **Presentation style:** Portal design with vs. without visual aids and explanatory text<br>- **Patient Health Engagement (PHE):** Measured pre- and post-exposure using a 5-item semantic differential scale assessing cognitive, emotional, and behavioral dimensions (adapted from Graffigna et al., 2015)	- Patient health engagement significantly declined after viewing abnormal or partially abnormal blood test results via the patient portal.<br>- Declines in PHE was significantly higher when test results were presented without explanatory text and visualizations.<br>- When normal or partially abnormal results included explanations and visuals, PHE remained stable.<br>- However, even with explanations and visuals, abnormal results still led to a significant decline in PHE.

Table 2. Detailed summary of reviewed studies

STUDY OVERVIEW (Citation, Study Design & Purpose)	POPULATION AND SAMPLE	Main Variables and Measures	MAIN FINDINGS
**Talboom-Kamp, Tossaint-Schoenmakers et al [30]**<br><br>This cross-sectional, real-world study investigated patient experiences and self-efficacy using an online patient portal that presents laboratory test results with explanatory text and visuals. Users who accessed their laboratory test results through a patient portal were invited to answer a web-based survey immediately after they viewed their test results. The study evaluated perceptions of usability and self-efficacy.	□ **Country**: Netherlands<br>□ **Eligibility criteria**: Patients who viewed their lab results on the Saltro patient portal between September 2018 and February 2019<br>□ **Survey participants** (n = 354); mean age = 58.5 years; 63% female	□ **Perceived usability**: Measured using the "Information and Presentation" subscale of the Dutch version of the eHealth Impact Questionnaire (eHIQ-2; Kelly et al., 2015); 5-point Likert scale.<br>□ **Self-efficacy**: Measured using the "Motivation and Confidence to Act" subscale of the eHIQ-2; 5-point Likert scale.	□ Participants gave high usability ratings, particularly for clarity of language, ease of understanding, and trust in the information.<br>□ The portal had a modest impact on patients' motivation and confidence to manage their health, especially with regard to using the portal to make health decisions, feeling confident discussing health with others, and explaining health concerns.<br>□ A significant positive correlation was found between perceived usability and self-efficacy ($r = .77$, $p < .001$); patients who found the portal easier to use and more trustworthy were also more confident and motivated to act on the information.
**Tossaint-Schoenmakers, Kasteleyn et al [31]**<br><br>This **cross-sectional, real-world study** examined how patient characteristics (age, gender, education, and chronic illness) influence users' perceptions of an online portal for accessing laboratory test results. Patients who viewed their blood test results via the portal were invited to complete a survey immediately afterward. The study focused on perceived usability and self-efficacy and used descriptive and regression analyses to examine associations with patient characteristics.	□ **Country**: Netherlands<br>□ **Eligibility criteria**: Patients who had blood tests and accessed results via a web-based portal<br>□ **Survey participants** (n = 748); mean age = 58.5 years (SD = 16.4); 57.2% female; 56.6% highly educated; 68% reported no chronic diseases. Chronic condition prevalence: diabetes (12.4%), asthma/COPD (7.2%), cardiovascular disease (11.6%).	□ **Perceived usability**: Measured with the "Information and Presentation" subscale of the Dutch version of the eHealth Impact Questionnaire (eHIQ-2; Kelly et al., 2015); 5-point Likert scale.<br>□ **Self-efficacy**: Measured with the "Motivation and Confidence to Act" subscale of the eHIQ-2; 5-point Likert scale.<br>□ **Patient characteristics**: Age, gender, education level, and presence of chronic disease (diabetes, asthma/COPD, cardiovascular disease).	□ Older age, higher education, and having asthma or COPD were associated with lower perceived usability of the portal.<br>□ Higher education was linked to lower self-efficacy in using test results to manage health.<br>□ No other demographic or clinical factors were significantly associated with self-efficacy ratings.
**Turer, Martin et al [32]**<br><br>This **retrospective observational study** examined real-time patient portal use among emergency department (ED) patients, focusing on whether and when	□ **Country:** United States<br>□ **Eligibility criteria:** Adult patients (≥18 years) with at least one test result during	□ **Patient portal use:** Whether patients or proxies viewed test results during the ED visit (binary variable based on portal audit logs)	□ The proportion of patients viewing results during ED visits increased from 5 to 15% over the study period, indicating an increase in lab portal use since implementation of the ONC's 21st Century *Cures Act Final Rule*.

Table 2. Detailed summary of reviewed studies

STUDY OVERVIEW (Citation, Study Design & Purpose)	POPULATION AND SAMPLE	Main Variables and Measures	MAIN FINDINGS
patients accessed their test results during their ED visit. Using audit log data from a single U.S. academic medical center (April 2021–April 2022), the study analyzed 60,314 ED encounters to determine the rate and predictors of portal use for viewing lab, imaging, or ECG results in real time. Logistic regression models were used to examine trends and identify demographic and social factors associated with portal engagement during the ED stay.	an emergency department (ED) visit<br>- **Data sources:** logs of 60,314 ED encounters representing 31,164 unique patients between April 5, 2021, and April 4, 2022.	- **Demographics:** Age, sex, race, ethnicity, primary language, and insurance type<br>- **Social vulnerability:** CDC's Social Vulnerability Index (SVI), capturing socioeconomic and demographic risk factors<br>- **Encounter characteristics**: Emergency Severity Index (ESI) triage score and visit disposition (e.g., admitted, discharged)	- Patients who were male, Black or Hispanic, Spanish-speaking, publicly insured or self-paid, or living in high social vulnerability areas were less likely to view results during ED encounters, while those admitted to the hospital and Asian or other race were more likely to access their results.<br>- With the exception of a brief mention in after-visit reports, portal enrollment during ED visits increased despite no active promotion.<br>- A temporary spike in real-time result viewing occurred immediately after a system change enabled default push notifications for released results, illustrating the behavioral impact of minor configuration changes.
**Wood, Pham et al [33]**<br><br>This study evaluated how switching to the immediate release of diagnostic test results affected the timeliness of patient viewing via a patient portal at a large academic medical center. Using a **retrospective pre-post design**, the researchers analyzed diagnostic test data from 204,605 patients across two 10-month periods—before and after implementation of the 21st Century Cures Act (February–December 2020 vs. February–December 2021). The analysis included both laboratory and imaging results accessed through Epic's MyChart portal.	- **Country:** United States<br>- **Eligibility criteria:** Logs of patients of all ages who had laboratory or imaging diagnostic tests between February 9, 2020, and December 9, 2021; patients with unknown or conflicting demographic data were excluded (n = 8,101).<br>- **Data sources:** Diagnostic tests (n = 3,809,397) from 204,605 unique patients; mean age = 44.3 years (SD = 23.6); 56.5% female; 84.1% White; 96.5% preferred English; 70.6% had an active MyChart account during the study period	- **Time to view results:** Whether diagnostic test results (lab or imaging) were viewed within 1 or 30 days after release; derived from portal audit log timestamps.<br>- **Portal activity:** Whether results were viewed by the patient or proxy through MyChart.<br>- **Demographic and contextual factors:** Age, sex, race (White vs. non-White), preferred language (English vs. not English), insurance type (private vs. public/uninsured), and care setting (outpatient, inpatient, emergency department).<br>- **Test type:** Whether the result was from a laboratory test (reference group) or imaging study.	- After immediate release was implemented, the percentage of patients who viewed test results within 1 day increased sharply—especially for lab results (22% to 31% for adults; 15% to 33% for pediatric proxy users).<br>- Most released results were laboratory tests, which accounted for the majority of increased portal use after the policy change.<br>- Weekend result release increased (3.2% pre-Act vs 15.3% post-Act), but viewing patterns remained consistent, with most views occurring on weekdays.<br>- Portal engagement was higher among White, female, English-speaking, and privately insured patients, particularly those seen in outpatient settings.<br>- Results were less likely to be viewed by patients who were older, non-White, non-English-speaking, used public insurance or were uninsured, or received care in emergency or inpatient settings.

Table 2. Detailed summary of reviewed studies

STUDY OVERVIEW (Citation, Study Design & Purpose)	POPULATION AND SAMPLE	Main Variables and Measures	MAIN FINDINGS
**Zhang, Citardi et al [34]**<br><br>This **sequential explanatory mixed-methods study** explored patients' experiences, needs, and challenges when interpreting lab test results through patient portals. A web-based survey (n = 203) was conducted to assess health literacy, technology proficiency, comprehension, emotional reactions, and portal preferences. Follow-up semi-structured interviews (n = 13) provided deeper insights into patients' interpretation difficulties and suggestions for improving portal usability and clarity. Quantitative data were analyzed descriptively and via correlation tests; qualitative data were thematically analyzed to uncover user perceptions, confusion points, and suggestions for improved portal design.	**Country:** United States<br>**Eligibility Criteria:** Participants were U.S. adults aged 18 or older who had previously accessed lab test results via a patient portal.<br>**Survey participants** (n = 203): Ages 18–80 years (most between 26–49); mean age not reported; 48.3% female; 69.5% White; 66.1% had a bachelor's degree or higher; 54.7% self-rated their health literacy above medium.<br>**Interview participants** (n = 13): Mean age not reported; 46% ages 26–49; 53.8% female.	**Health literacy:** 1-item self-rating on a 5-point Likert scale ("Please rate your level of health literacy.")<br>**Technology proficiency:** 1-item self-rating on a 5-point Likert scale ("Please rate your technology proficiency.")<br>**Comprehension:** Perceived understanding of test results; yes/no/unsure responses (e.g., "Did you understand the result?").<br>**Emotional response:** 1-item question ("How did you feel when you saw the result?") with 3 options (positive, negative, indifferent).<br>**Information needs:** Multiple-choice items on what participants wanted to know (e.g., treatment, prognosis, next steps, reference ranges).<br>**Perceived usability/usefulness:** 5 items using a 3-point Likert scale (Agree / Neither / Disagree); e.g., "I am comfortable using patient portals to review my lab results."<br>**Portal feature preferences:** Multiple-choice list of five desired features (e.g., clearer presentation, trusted sources, easier navigation).	Sixty percent of survey respondents reported difficulty understanding their lab results, primarily due to medical jargon, unclear reference ranges, confusion about what values were normal or abnormal, and difficulty interpreting the overall meaning of the results.<br>Most participants said they needed more information—such as treatment options, prognosis, or questions to ask—after viewing their results.<br>The most common follow-up actions were searching online (62%) and consulting family or friends (50%), with relatively few contacting their doctor directly.<br>Higher technology proficiency was linked to greater perceived ease and usefulness of the portal; health literacy was associated with use of embedded resources and the desire for timely explanations and follow-up guidance.<br>Patients receiving abnormal results reported increased anxiety and a stronger need for both emotional and informational support.<br>Participants suggested that timely result explanations, access to a health encyclopedia, and secure messaging with providers would improve comprehension.<br>Additional recommendations included simplifying language, enabling visual tracking of past results, and enhancing accessibility for older adults and users with disabilities.<br>Some participants proposed integrating AI to provide personalized explanations based on individual health data.
**Zhang, Kmoth et al [35]**<br><br>This **user-centered, mixed-methods design study** aimed to develop and	**Country**: United States<br>**Eligibility Criteria**: Adults who had recently accessed	**Lab result comprehension barriers**: Confusion interpreting	Participants valued tailored explanations, contextualized next steps, and support resources based on their medical situation.

Table 2. Detailed summary of reviewed studies

STUDY OVERVIEW (Citation, Study Design & Purpose)	POPULATION AND SAMPLE	Main Variables and Measures	MAIN FINDINGS
evaluate a patient-facing system prototype that improves the communication of laboratory test results. The study combined a survey (n=203) and interviews (n=13) to identify patient challenges, informational needs, and technological preferences when interpreting test results. These insights guided the iterative design of an interactive prototype, which included features such as graphical displays, plain-language explanations, question-prompting tools, and contextualized sources. A pilot evaluation involving 8 participants assessed the prototype's usability and perceived usefulness using think-aloud protocols and follow-up interviews.	lab test results through a patient portal<br>- **Survey participants** (n = 203): ages 18–80 years (mean = 43.5); 61.6% female; 71.9% White<br>- **Interview participants** (n = 13): ages 20–68 years (mean = 45.9); 61.5% female; 69.2% White	values, medical jargon, and missing follow-up guidance<br>- **Information needs**: Desire for general test explanations and personalized information about health implications or next steps<br>- **Technology support preferences**: Interest in graphical summaries, clickable definitions, trusted sources, tailored info, and annotation tools<br>- **Usability**: 10-item System Usability Scale (SUS) with 5-point response options (Brooke, 1996; e.g., "I thought the system was easy to use.")<br>- **Feature ratings**: Perceived usefulness of individual components, such as the graphical display, Ask Questions section, and annotation functions	- Automated highlighting and plain-language definitions of medical terms were rated as highly useful.<br>- The "Ask Questions" feature, which provided patient-specific prompts, was considered one of the most helpful components.<br>- Some participants questioned the credibility of information and preferred sources from trusted institutions or peer-reviewed journals.<br>- Emotionally sensitive wording (e.g., "not optimal") triggered anxiety; participants emphasized the need for empathetic design.<br>- Participants recommended tailoring explanations to the user's health literacy level, offering both basic summaries and advanced content.<br>- Support was expressed for both mobile and web versions to accommodate different use contexts, such as clinic visits vs. at-home review.
**Zhong, Park et al [36]**<br><br>This **retrospective study** explored how different patterns of patient portal use—specifically lab results, messaging, medication information, and appointment management—related to changes in healthcare utilization and appointment adherence. Audit log data from patient portals were linked to EMR records and analyzed over a 2.5-year period (2013–2016). Propensity score–matched groups of users and nonusers were compared to evaluate visit frequency and missed appointments over time.	- **Country**: United States<br>- **Eligibility Criteria:** Adult primary care patients (aged 18+) seen at University of Florida Health between 2013 and 2016.<br>- **Data sources:** logs of an initial cohort of 46,544 patients, matched groups were analyzed:<br>- **Portal users** (n = 4,312): 35.2% aged 46–64; 28.6% aged 31–45; 65.8% female; 74.1% White<br>- **Matched nonusers** (n = 4,024): 33.9% aged 46–64;	- **Portal feature use**: Frequency of access to messaging, lab results, medication lists, and appointment tools; categorized by audit log data<br>- **Healthcare engagement**: Number of primary care office visits and telephone encounters<br>- **Appointment adherence**: No-show and cancellation rates<br>- **Patient characteristics**: Age, sex, race/ethnicity, insurance type, and health status (measured by Active Problem Number [APN] as proxy for disease burden)	- Lab results were one of the most frequently accessed portal features, used by 85% of portal adopters.<br>- Patients who used both lab results and messaging features had significantly more office visits and telephone encounters in the two quarters after portal adoption compared to matched controls.<br>- Lab and messaging users had 30–60% lower appointment no-show rates across multiple quarters.<br>- Portal adoption was significantly lower among Black, Hispanic, unmarried, and publicly insured patients, and higher among White, female, married, and privately insured users.

Table 2. Detailed summary of reviewed studies

STUDY OVERVIEW (Citation, Study Design & Purpose)	POPULATION AND SAMPLE	Main Variables and Measures	MAIN FINDINGS
	29.4% aged 31–45; 64.9% female; 74.1% White		☐ More than one-third of portal adopters became inactive for at least one quarter, indicating inconsistent engagement even among those who enrolled.
**Zikmund-Fisher, Exe et al [37]**<br><br>This **online experiment** tested whether adults with and without diabetes could identify out-of-range values when viewing standard tabular lab results, similar to those found in patient portals. The study also examined how health literacy, numeracy, and prior experience with diabetes affected comprehension, perceived urgency, and behavioral intentions. Participants (N = 1,817) were randomly assigned in a 2×2 factorial design:<br><br>☐ Hemoglobin A1c value: moderately elevated (7.1%) or substantially elevated (8.4%)<br>☐ Single deviation (where only A1c was out of range); or multiple deviations (A1c and several other tests were abnormal)<br>All participants viewed results in the same standard tabular format without visual cues or interpretive aids.	☐ **Country:** United States<br>☐ **Eligibility Criteria:** Adults recruited from a national online panel of internet users (Survey Sampling International)<br>☐ **Participants** (n = 1,817); average age = 54.2 years; 50.5% female; 77.7% White non-Hispanic; **53.6%** reported having diabetes**.**	☐ **Comprehension**: Accuracy in identifying values outside the standard range<br>☐ **Behavioral intentions**: Intentions to contact a doctor in response to the results<br>☐ **Perceived blood glucose control**: 11-point scale assessing perception about how well blood sugar levels are under control.<br>☐ **Health literacy**: 3-item Chew scale (e.g., "How confident are you filling out medical forms?"), 5-point Likert scale<br>☐ **Numeracy**: 8-item Subjective Numeracy Scale (SNS; Fagerlin et al., 2007), 6-point agreement scale (e.g., "How good are you at calculating a 15% tip?")<br>☐ **Perceived usefulness:** Self-reported understanding and helpfulness of the test results using 5-point scales	☐ Only 51% correctly identified a hemoglobin A1c value of 8.4% as out of range.<br>☐ Participants shown multiple test deviations were significantly more likely to flag 8.4% as out of range than those in the single deviation condition.<br>☐ Among diabetes patients, higher health literacy was associated with lower likelihood of calling a doctor for mildly elevated A1c (7.1%), while higher numeracy predicted greater likelihood of contacting a provider for more elevated A1c (8.4%).<br>☐ Non-diabetic participants in the multiple deviation condition were more likely to call for mild elevations, but less likely to call for substantial elevations.<br>☐ Perceived blood glucose control was significantly associated with correct interpretation of A1c levels.<br>☐ Many participants rated the standard table displays as difficult to understand or not useful.
**Zikmund-Fisher, Scherer et al [38]**<br><br>This **experimental study** evaluated whether visual displays (number line formats) improved patients' ability to distinguish between mildly and severely abnormal lab test results compared to standard tabular displays. Participants were shown fictional lab results (platelet	☐ **Country**: United States<br>☐ **Eligibility Criteria**: Adults aged 18+, recruited through an online panel with quotas for age and race to mirror U.S. census demographics.<br>☐ **Participant Characteristics**: Participants (n = 1,620);	☐ **Perceived Urgency**: Measured using 2 items ("How alarming does this result feel?" and "How urgent is it to take action?"), averaged on a 6-point scale; high internal consistency (α = 0.91–0.95)<br>☐ **Behavioral Intentions**: Multiple-choice question asking how	☐ Participants who viewed **tables** were significantly **more likely to overestimate the urgency** of mildly abnormal results compared to those shown **visual displays** (simple line, color blocks, or gradient line).<br>☐ **Gradient line displays** helped participants **distinguish more accurately** between mildly and severely abnormal results.

Table 2. Detailed summary of reviewed studies

STUDY OVERVIEW (Citation, Study Design & Purpose)	POPULATION AND SAMPLE	Main Variables and Measures	MAIN FINDINGS
count, ALT, and serum creatinine) in one of four formats: a table, a simple number line, a color-block line, or a gradient line. The researchers assessed participants' perceived urgency, behavioral intentions, and display preferences.	average age = 48.9 years; 52.3% female; 77.4% White. Health literacy (mean = 4.24/5), numeracy (mean = 4.47/6), and graphical literacy (mean = 3.76/6) scores were also collected.	participants would respond to each result (e.g., do nothing, wait, call doctor, go to the hospital)<br>☐ **Display Preferences**: Rated on 4 items (trust, clarity, helpfulness, desire to see again) using a 5-point Likert scale; α = 0.88<br>☐ **Health Literacy**: 1-item screener (Chew et al.): "How confident are you filling out medical forms by yourself?" (1 = not at all, 5 = extremely confident)<br>☐ **Subjective Numeracy**: 8-item Subjective Numeracy Scale (SNS; Fagerlin et al., 2007), 6-point agreement scale; assesses perceived ability and preference for numerical information (e.g., "How good are you at calculating percentages?")<br>☐ **Graphical Literacy**: 6-item test (Galesic & Garcia-Retamero, 2011), scored as number of correct responses to graph-based questions	☐ Visual formats—especially gradient and block displays—were rated as more helpful and easier to interpret than tables.<br>☐ Participants who viewed visual formats were **less likely to say they would immediately contact a provider** for near-normal results, suggesting reduced unnecessary follow-up.<br>☐ Participants with higher graphical or health literacy had greater accuracy in urgency judgments, but even those with lower skills benefited from the gradient display.
**Zikmund-Fisher, Scherer et al [39]**<br><br>This **experimental study** explored whether adding a "harm anchor"—a visual marker that communicates when doctors typically become concerned—could reduce patients' perceived urgency, worry, and unnecessary reactions to mildly abnormal test results. Participants were randomly assigned to view ALT, creatinine, and platelet count results presented in one of four visual formats: a simple line,	☐ **Country**: United States<br>☐ **Eligibility Criteria**: Adults aged 18 years or older recruited through a U.S.-based national internet panel (Survey Sampling International)<br>☐ **Participants** (n = 1,618); ages 19–89 years (mean = 48.8); 52.1% female; 77.8% White. Participants reflected a broad range of	☐ **Display format**: Simple line, color blocks, gradient, or harm anchor with label (e.g., "many doctors are not concerned until here")<br>☐ **Perceived urgency and concern**: Two 6-point Likert items ("How alarming does this result feel to you?" and "How urgent is it to take action based on this result?"), averaged; α = .91–.95<br>☐ **Behavioral intentions**: Response options for how they would act	☐ Participants who viewed the harm anchor display rated near-normal ALT and creatinine results as less urgent than those shown other formats; urgency ratings for extreme values did not differ across formats.<br>☐ The harm anchor display improved participants' ability to differentiate between near-normal and more concerning results in terms of urgency and behavioral intentions.<br>☐ Participants who saw the harm anchor format were less likely to say they would call their doctor or go to the hospital for near-normal

Table 2. Detailed summary of reviewed studies

STUDY OVERVIEW (Citation, Study Design & Purpose)	POPULATION AND SAMPLE	Main Variables and Measures	MAIN FINDINGS
color-coded blocks, gradient lines, or a harm anchor line. After viewing the results, participants rated their emotional reactions, perceived urgency, behavioral intentions, and overall format preference.	health literacy and numeracy levels.	(e.g., call doctor immediately, wait, or do nothing)<br>• **Display preference**: Composite score across four 5-point Likert items (e.g., trust, helpfulness, clarity); α = .87<br>• **Demographic characteristics**: Age, gender, race/ethnicity, health literacy, subjective numeracy (used to test for response differences)	results and more likely to wait or take no action.<br>• Despite these effects on urgency perception and intended follow-up behavior, participants rated the harm anchor display similarly to other formats in terms of trust, helpfulness, and preference.

REFERENCES

1. Bhalla S, Prasad T, Xie D, Gerber DE. Contemporary trends in reviewing test results through the electronic patient portal among patients with cancer. JAMA Oncol. 2024 Jan 1;10(1):139–40. PMID: 38032648. doi: 10.1001/jamaoncol.2023.5047.

2. Christensen K, Sue V. Viewing laboratory test results online: Patients' actions and reactions. J Participat Med. 2013 Oct. 3;5:e38.

3. Foster B, Krasowski MD. The use of an electronic health record patient portal to access diagnostic test results by emergency patients at an academic medical center: Retrospective study. J Med Internet Res. 2019 Jun 28;21(6):e13791. PMID: 31254335. doi: 10.2196/13791.

4. Fraccaro P, Vigo M, Balatsoukas P, van der Veer SN, Hassan L, Williams R, et al. Presentation of laboratory test results in patient portals: Influence of interface design on risk interpretation and visual search behaviour. BMC Med Inform Decis Mak. 2018 Feb 12;18(1):11. PMID: 29433495. doi: 10.1186/s12911-018-0589-7.

5. Giardina TD, Modi V, Parrish DE, Singh H. The patient portal and abnormal test results: An exploratory study of patient experiences. Patient Exp J. 2015 Spring;2(1):148–54. PMID: 28345018.

6. Giardina TD, Baldwin J, Nystrom DT, Sittig DF, Singh H. Patient perceptions of receiving test results via online portals: A mixed-methods study. J Am Med Inform Assoc. 2018 Apr 1;25(4):440–6. PMID: 29240899. doi: 10.1093/jamia/ocx140.

7. Hulter P, Langendoen W, Pluut B, Schoonman GG, Luijten R, van Wetten F, et al. Patients' choices regarding online access to laboratory, radiology and pathology test results on a hospital patient portal. PloS One. 2023;18(2):e0280768. PMID: 36735739. doi: 10.1371/journal.pone.0280768.

8. Hulter P, Weggelaar-Jansen A, Ahaus K, Pluut B. Patient discourses on real-time access to test results via hospital portals: A discourse analysis of semistructured interviews with Dutch patients. BMJ Open. 2024 Nov 24;14(11):e088201. PMID: 39581732. doi: 10.1136/bmjopen-2024-088201.

9. Joseph AL, Monkman H, MacDonald L, Lai C. Interpreting laboratory results with complementary health information: A human factors perspective. Stud Health Technol Inform. 2024 Jan 25;310:1061–5. PMID: 38269977. doi: 10.3233/SHTI231127.

10. Krasowski MD, Grieme CV, Cassady B, Dreyer NR, Wanat KA, Hightower M, et al. Variation in results release and patient portal access to diagnostic test results at an academic medical center. J Pathol Inform. 2017 2017/01/01/;8(1):45. PMID: 29226008. doi: 10.4103/jpi.jpi_53_17.

11. Lustria MLA, Aliche O, Killian MO, He Z. Enhancing patient engagement and understanding: Is providing direct access to laboratory results through patient portals adequate? JAMIA Open. 2025 Apr 2025;8(2):ooaf009. PMID: 40130170. doi: 10.1093/jamiaopen/ooaf009.

12. Mak G, Smith Fowler H, Leaver C, Hagens S, Zelmer J. The effects of web-based patient access to laboratory results in British Columbia: A patient survey on comprehension and anxiety. J Med Internet Res. 2015 Aug 4;17(8):e191. PMID: 26242801. doi: 10.2196/jmir.4350.

13. McFarland JA, Huang J, Li Y, Gunn AJ, Morgan DE. Patient engagement with online portals and online radiology results. Curr Probl Diagn Radiol. 2023 Mar–Apr;52(2):106–9. PMID: 36030140. doi: 10.1067/j.cpradiol.2022.07.012.

14. Monkman H, Griffith J, MacDonald L, Joseph AL, Lesselroth B. Why do people use online lab results and what do they look for: A qualitative study. Stud Health Technol Inform. 2022;294:599–603. doi: 10.3233/shti220539.

15. Monkman H, MacDonald L, Nohr C, Tanaka JW, Lesselroth BJ. Hidden in plain sight: Overlooked results and other errors in evaluating online laboratory results. Stud Health Technol Inform. 2022 Jun 6;290:867–71. PMID: 35673142. doi: 10.3233/SHTI220203.

16. Monkman H, Griffith J, MacDonald L, Lesselroth B. Consumers' needs for laboratory results portals: Questionnaire study. JMIR Hum Factors. 2023 Jun 12;10:e42843. PMID: 37307049. doi: 10.2196/42843.

17. Monkman H, MacDonald L, Joseph AL, Lesselroth BJ. Tabular, annotated, visual, or trends + contextual information? Preferences for online laboratory results displays. Stud Health Technol Inform. 2024 Jan 25;310:1041–5. PMID: 38269973. doi: 10.3233/SHTI231123.

18. Monkman H, Schmit A, Nyholt D, MacDonald L, Lesselroth B. False calm and false alarm: A qualitative study of confusion and misinterpretation of a laboratory results graph. Stud Health Technol Inform. 2025 May 12;326:106–10. PMID: 40357611. doi: 10.3233/SHTI250248.

19. Morrow D, Azevedo RFL, Garcia-Retamero R, Hasegawa-Johnson M, Huang T, Schuh W, et al. Contextualizing numeric clinical test results for gist comprehension: Implications for EHR patient portals. J Exp Psychol Appl. 2019 Mar;25(1):41–61. PMID: 30688498. doi: 10.1037/xap0000203.

20. Nystrom DT, Singh H, Baldwin J, Sittig DF, Giardina TD. Methods for patient-centered interface design of test result display in online portals. EGEMS (Wash DC). 2018 Jun 26;6(1):15. PMID: 30094287. doi: 10.5334/egems.255.

21. Pillemer F, Price RA, Paone S, Martich GD, Albert S, Haidari L, et al. Direct release of test results to patients increases patient engagement and utilization of care. PloS One. 2016;11(6):e0154743. PMID: 27337092. doi: 10.1371/journal.pone.0154743.

22. Robinson S, Reed M, Quevillon T, Hirvi E. Patient perceptions and interactions with their web portal-based laboratory results. BMJ Health Care Inform. 2019 Apr;26(1):0. PMID: 31039117. doi: 10.1136/bmjhci-2019-000012.

23. Scherer AM, Witteman HO, Solomon J, Exe NL, Fagerlin A, Zikmund-Fisher BJ. Improving the understanding of test results by substituting (not adding) goal ranges: Web-based between-subjects experiment. J Med Internet Res. 2018 Oct 19;20(10):e11027. PMID: 30341053. doi: 10.2196/11027.

24. Schultz CL, Alderfer MA. Are on-line patient portals meeting test result preferences of caregivers of children with cancer? A qualitative exploration. Pediatr Blood Cancer. 2018 Nov;65(11):e27306. PMID: 30007016. doi: 10.1002/pbc.27306.

25. Solomon J, Scherer AM, Exe NL, Witteman HO, Fagerlin A, Zikmund-Fisher BJ, editors. Is this good or bad? Redesigning visual displays of medical test results in patient portals to provide context and meaning. 2016 CHI Conference Extended Abstracts on Human Factors in Computing Systems; 2016; San Jose, California, USA: Association for Computing Machinery.

26. Steitz BD, Turer RW, Lin CT, MacDonald S, Salmi L, Wright A, et al. Perspectives of patients about immediate access to test results through an online patient portal. JAMA Netw Open. 2023 Mar 1;6(3):e233572. PMID: 36939703. doi: 10.1001/jamanetworkopen.2023.3572.

27. Steitz BD, Turer RW, Salmi L, Suresh U, MacDonald S, DesRoches CM, et al. Repeated access to patient portal while awaiting test results and patient-initiated messaging. JAMA Netw Open. 2025 Apr 1;8(4):e254019. PMID: 40198070. doi: 10.1001/jamanetworkopen.2025.4019.

28. Steitz BD, Guide A, Rodriguez K, Kripalani S, Aher CV, Craig KS, et al. Patient-friendly test results and patient-initiated messaging among adult outpatients. JAMA Network Open. 2025;8(11):e2543879–e. doi: 10.1001/jamanetworkopen.2025.43879.

29. Struikman B, Bol N, Goedhart A, van Weert JCM, Talboom-Kamp E, van Delft S, et al. Features of a patient portal for blood test results and patient health engagement: Web-based pre-post experiment. J Med Internet Res. 2020 Jul 20;22(7):e15798. PMID: 32706704. doi: 10.2196/15798.

30. Talboom-Kamp E, Tossaint-Schoenmakers R, Goedhart A, Versluis A, Kasteleyn M. Patients' attitudes toward an online patient portal for communicating laboratory test results: Real-world study using the eHealth impact questionnaire. JMIR Form Res. 2020 Mar 4;4(3):e17060. PMID: 32024632. doi: 10.2196/17060.

31. Tossaint-Schoenmakers R, Kasteleyn M, Goedhart A, Versluis A, Talboom-Kamp E. The impact of patient characteristics on their attitudes toward an online patient portal for communicating laboratory test results: Real-world study. JMIR Form Res. 2021 Dec 17;5(12):e25498. PMID: 34927593. doi: 10.2196/25498.

32. Turer RW, Martin KR, Courtney DM, Diercks DB, Chu L, Willett DL, et al. Real-time patient portal use among emergency department patients: An open results study. Appl Clin Inform. 2022 Oct;13(5):1123–30. PMID: 36167337. doi: 10.1055/a-1951-3268.

33. Wood KE, Pham HT, Carter KD, Nepple KG, Blum JM, Krasowski MD. Impact of a switch to immediate release on the patient viewing of diagnostic test results in an online portal at an academic medical center. J Pathol Inform. 2023 2023/01/01/;14:100323. PMID: 37520309. doi: 10.1016/j.jpi.2023.100323.

34. Zhang Z, Citardi D, Xing A, Luo X, Lu Y, He Z. Patient challenges and needs in comprehending laboratory test results: Mixed methods study. J Med Internet Res. 2020 Dec 7;22(12):e18725. PMID: 33284117. doi: 10.2196/18725.

35. Zhang Z, Kmoth L, Luo X, He Z. User-centered system design for communicating clinical laboratory test results: Design and evaluation study. JMIR Hum Factors. 2021 Nov 25;8(4):e26017. PMID: 34842529. doi: 10.2196/26017.

36. Zhong X, Park J, Liang M, Shi F, Budd PR, Sprague JL, et al. Characteristics of patients using different patient portal functions and the impact on primary care service utilization and appointment adherence: Retrospective observational study. J Med Internet Res. 2020 Feb 25;22(2):e14410. PMID: 32130124. doi: 10.2196/14410.

37. Zikmund-Fisher BJ, Exe NL, Witteman HO. Numeracy and literacy independently predict patients' ability to identify out-of-range test results. J Med Internet Res. 2014 Aug 8;16(8):e187. PMID: 25135688. doi: 10.2196/jmir.3241.

38. Zikmund-Fisher BJ, Scherer AM, Witteman HO, Solomon JB, Exe NL, Tarini BA, et al. Graphics help patients distinguish between urgent and non-urgent deviations in laboratory test results. J Am Med Inform Assoc. 2017 May 1;24(3):520–8. PMID: 28040686. doi: 10.1093/jamia/ocw169.

39. Zikmund-Fisher BJ, Scherer AM, Witteman HO, Solomon JB, Exe NL, Fagerlin A. Effect of harm anchors in visual displays of test results on patient perceptions of urgency about near-normal values: Experimental study. J Med Internet Res. 2018 Mar 26;20(3):e98. PMID: 29581088. doi: 10.2196/jmir.8889.
