# Supplementary material for: Patient Experiences With Online Laboratory Test Presentations From Access to Activation: Systematic Review
Source: J Med Internet Res. 2026 May 29;28:e88259. doi: 10.2196/88259 (PMC13222930; doi:10.2196/88259)
Supplement: Checklist 2 [file jmir-v28-e88259-s004.pdf]

CHECKLIST 2 – SWiM Checklist

Synthesis Without Meta-analysis (SWiM) Reporting Items

SWiM reporting item	Item description	Page in manuscript where item is reported
Methods		
**1** Grouping studies for synthesis	1a) Provide a description of, and rationale for, the groups used in the synthesis (e.g., groupings of populations, interventions, outcomes, study design)	Methods – Data Extraction and Synthesis (Analysis Strategy; Coding and Thematic Synthesis)
	1b) Detail and provide rationale for any changes made subsequent to the protocol in the groups used in the synthesis	Methods – Protocol and Registration
**2** Describe the standardised metric and transformation methods used	Describe the standardised metric for each outcome. Explain why the metric(s) was chosen, and describe any methods used to transform the intervention effects, as reported in the study, to the standardised metric, citing any methodological guidance consulted	Not applicable – Narrative synthesis without effect size transformation
**3** Describe the synthesis methods	Describe and justify the methods used to synthesise the effects for each outcome when it was not possible to undertake a meta-analysis of effect estimates	Methods – Analysis Strategy
**4** Criteria used to prioritise results for summary and synthesis	Where applicable, provide the criteria used, with supporting justification, to select the particular studies, or a particular study, for the main synthesis or to draw conclusions from the synthesis (e.g., based on study design, risk of bias assessments, directness in relation to the review question)	Methods – Data Extraction and Synthesis; Methods – Quality Assessment (p. 7-8); Multimedia Appendix 1 – Risk of Bias Tables 1-5
**5** Investigation of heterogeneity in reported effects	State the method(s) used to examine heterogeneity in reported effects when it was not possible to undertake a meta-analysis of effect estimates and its extensions to investigate heterogeneity	Methods – Analysis Strategy; Results – Results of Thematic Analysis
**6** Certainty of evidence	Describe the methods used to assess certainty of the synthesis findings	Methods – Analysis Strategy
**7** Data presentation methods	Describe the graphical and tabular methods used to present the effects (e.g., tables, forest plots, harvest plots).<br><br>Specify key study characteristics (e.g., study design, risk of bias) used to order the studies, in the text and any tables or graphs, clearly referencing the studies included	Results (Table 1); Multimedia Appendix 1 – Risk of Bias Tables 1-5; Multimedia Appendix 2 – Detailed Summary of Included Studies
Results		

SWiM reporting item	Item description	Page in manuscript where item is reported
**8** Reporting results	For each comparison and outcome, provide a description of the synthesised findings, and the certainty of the findings. Describe the result in language that is consistent with the question the synthesis addresses, and indicate which studies contribute to the synthesis	Results – Results of Thematic Analysis
Discussion		
**9** Limitations of the synthesis	Report the limitations of the synthesis methods used and/or the groupings used in the synthesis, and how these affect the conclusions that can be drawn in relation to the original review question	Discussion – Limitations

From: Campbell M, McKenzie JE, Sowden A, Katikireddi SV, Brennan SE, Ellis S, et al. Synthesis without meta-analysis (SWiM) in systematic reviews: Reporting guideline. BMJ. 2020 Jan 16;368:l6890. PMID: 31948937. doi: 10.1136/bmj.l6890.
